# A new framework for host-pathogen interaction research

**DOI:** 10.3389/fimmu.2022.1066733

**Published:** 2022-12-15

**Authors:** Hong Yu, Li Li, Anthony Huffman, John Beverley, Junguk Hur, Eric Merrell, Hsin-hui Huang, Yang Wang, Yingtong Liu, Edison Ong, Liang Cheng, Tao Zeng, Jingsong Zhang, Pengpai Li, Zhiping Liu, Zhigang Wang, Xiangyan Zhang, Xianwei Ye, Samuel K. Handelman, Jonathan Sexton, Kathryn Eaton, Gerry Higgins, Gilbert S. Omenn, Brian Athey, Barry Smith, Luonan Chen, Yongqun He

**Affiliations:** ^1^ Department of Respiratory and Critical Care Medicine, Guizhou Provincial People’s Hospital and National Health Commission (NHC) Key Laboratory of Immunological Diseases, People’s Hospital of Guizhou Province, Guiyang, Guizhou, China; ^2^ Department of Basic Medicine, Guizhou University Medical College, Guiyang, Guizhou, China; ^3^ Department of Genetics, Harvard Medical School, Boston, MA, United States; ^4^ University of Michigan Medical School, Ann Arbor, MI, United States; ^5^ Department of Philosophy, University at Buffalo, Buffalo, NY, United States; ^6^ Asymmetric Operations Sector, Johns Hopkins University Applied Physics Laboratory, Laurel, MD, United States; ^7^ Department of Biomedical Sciences, University of North Dakota School of Medicine and Health Sciences, Grand Forks, ND, United States; ^8^ Department of Biotechnology and Laboratory Science in Medicine, National Yang-Ming University, Taipei, Taiwan; ^9^ Department of Bioinformatics, Harbin Medical University, Harbin, Helongjian, China; ^10^ Key Laboratory of Systems Biology, Center for Excellence in Molecular Cell Science, Shanghai Institute of Biochemistry and Cell Biology, Chinese Academy of Sciences, Shanghai, China; ^11^ Center of Intelligent Medicine, School of Control Science and Engineering, Shandong University, Jinan, Shandong, China; ^12^ Department of Biomedical Engineering, Institute of Basic Medical Sciences and School of Basic Medicine, Peking Union Medical College and Chinese Academy of Medical Sciences, Beijing, China

**Keywords:** host-pathogen interaction, disease outcome, COVID-19, host-coronavirus interaction, coronavirus infectious disease ontology (CIDO), bioinformatics, COVID-19 cocktail, HPIPO framework

## Abstract

COVID-19 often manifests with different outcomes in different patients, highlighting the complexity of the host-pathogen interactions involved in manifestations of the disease at the molecular and cellular levels. In this paper, we propose a set of postulates and a framework for systematically understanding complex molecular host-pathogen interaction networks. Specifically, we first propose four host-pathogen interaction (HPI) postulates as the basis for understanding molecular and cellular host-pathogen interactions and their relations to disease outcomes. These four postulates cover the evolutionary dispositions involved in HPIs, the dynamic nature of HPI outcomes, roles that HPI components may occupy leading to such outcomes, and HPI checkpoints that are critical for specific disease outcomes. Based on these postulates, an HPI Postulate and Ontology (HPIPO) framework is proposed to apply interoperable ontologies to systematically model and represent various granular details and knowledge within the scope of the HPI postulates, in a way that will support AI-ready data standardization, sharing, integration, and analysis. As a demonstration, the HPI postulates and the HPIPO framework were applied to study COVID-19 with the Coronavirus Infectious Disease Ontology (CIDO), leading to a novel approach to rational design of drug/vaccine cocktails aimed at interrupting processes occurring at critical host-coronavirus interaction checkpoints. Furthermore, the host-coronavirus protein-protein interactions (PPIs) relevant to COVID-19 were predicted and evaluated based on prior knowledge of curated PPIs and domain-domain interactions, and how such studies can be further explored with the HPI postulates and the HPIPO framework is discussed.

## Introduction

Effectively combatting infectious diseases such as COVID-19 requires identifying the underlying causal molecular mechanisms that drive disease outcomes. It is not enough to know whether a patient has an infection or whether they exhibit signs and symptoms of a disease. Similar infections can result in distinct diseases, and identical diseases can, in turn, produce distinct signs and symptoms. Focusing on pathogens at the exclusion of hosts, or hosts at the exclusion of pathogens, is also insufficient for identifying mechanisms driving disease outcomes. Pathogen-centered approaches often struggle to isolate specific pathogens, such as *Candida albicans* and *Staphylococcus aureus*, that may co-colonize both symptomatic and asymptomatic hosts (1). Host-centered approaches fill some gaps in pathogen-centered strategies, but face difficulties in accommodating pathogens like *Staphylococcus epidemidis* which do not cause disease in all hosts. A complete picture of pathogenesis will balance consideration of both host *and* pathogen.

One step towards synthesizing host-centered and pathogen-centered approaches is The Damage Response Framework (DRF), proposed by Casadevall and Pirofski in 2003 (2). The DRF provides a high-level classification of pathogens based on host-pathogen interactions (HPIs) and rests on three tenets: (i) Microbial pathogenesis is an outcome of an interaction between a host and a microorganism. (ii) The host-relevant outcome of the host–microorganism interaction is determined by the amount of damage to the host. (iii) Host damage can result from both microbial factors and host response (2). However, the DRF is not fine-grained enough to identify *causal mechanisms* of disease outcomes at the molecular level. Consequently, it is unclear how exactly to understand “damage” in the DRF. What seems needed is a framework for investigating mechanisms of disease outcomes at the *molecular* level that – like DRF – emphasizes contributions from both host and pathogen to varied disease outcomes.

We propose four HPI postulates to take a further step towards a molecular level synthesis of host-centered and pathogen-centered approaches to understanding and treating disease. These postulates are intended to facilitate understanding of complex molecular mechanisms involved in HPIs. That said, researchers investigating infectious diseases are met with another challenge in the presence of the continuously growing stores of multidimensional, complex data. Perrin-Cocon et al., for example, recently assembled a large host-coronavirus interactome representation covering 1,311 protein-protein interactions (PPIs) documented in the literature (3). Similarly, Gordon et al. performed comparative viral-human PPI and viral protein localization analyses for SARS-CoV-1, SARS-CoV-2 and MERS-CoV and found pan-viral disease mechanisms across all three viruses (4). Such infectious disease data is often heterogeneous, poorly integrated, and non-interoperable, which prevents computer-assisted discovery, reasoning, and analysis, especially because so many different components and pathways are involved in different host-coronavirus interaction processes (5). Fortunately, these problems can be addressed through the use of carefully developed ontologies.

Ontologies are controlled, structured vocabularies representing entities and the relations among them. For two decades, ontologies have played an important role in standardizing the representation, integration, discovery, sharing, and analysis of knowledge and data (6–12). A major field of artificial intelligence (AI) research is knowledge representation and reasoning (KR², or KR&R), of which ontology is a foundational discipline. In the early years of KR&R, small ontologies were developed. In the era of big data, interoperable ontologies are needed to support big data standardization and integration (13–16). Interoperable ontologies are also critical to support data FAIRness, i.e., having data Findable, Accessible, Interoperable and Reusable (17, 18). Significant progress has been made in the construction of ontologies covering pathogens, hosts, and the molecular constituents of associated mechanisms of interaction. As an example, we recently developed and employed the Coronavirus Infectious Disease Ontology (CIDO) (19–21) to identify potential treatment options based on an analysis of molecular mechanisms identified as being linked with specific disease outcomes (22).

Given the successes of ontologies in addressing big data problems in the biomedical domain, we chose to implement our HPI postulates using ontologies. The HPI postulates may be implemented using alternative strategies, but for our purposes we implement the postulates in what we will call the Host-Pathogen Interaction Postulates and Ontology (HPIPO) framework. Doing so ensures the data generated through the application of the HPI postulates will remain well-organized and interoperable across a wide range of existing ontologies. In what follows we present the HPI postulates and illustrate how they can be used to incorporate host centered and pathogen centered approaches to the understanding of disease outcomes. We also show how the Postulates and applied ontologies come together in the HPIPO framework, using the CIDO to represent COVID-19 outcomes.

## Fundamental Host-Pathogen Interaction Postulates

**Figure 1:**
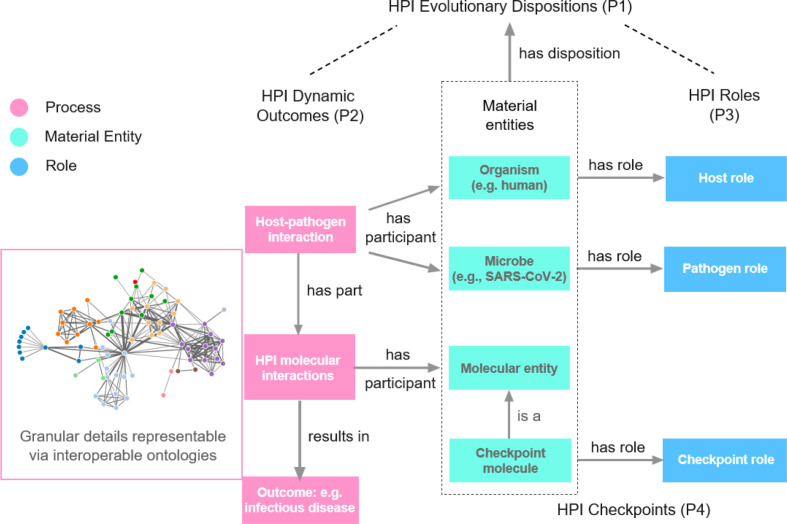
HPI Postulates. The network image is a symbol of the resulting huge interaction network we usually see. To better study such networks in host-pathogen interactions (HPIs) and the resulting disease outcomes, we propose four HPI postulates (HPIP), including HPI evolutionary dispositions (P1), HPI dynamic outcomes (P2), HPI roles (P3), and HPI checkpoints (P4), as the basic framework. The HPI postulates suggest the identification and definition of the roles of different nodes and edges in the network and how they are related to disease outcomes. Ontology supports such integrative knowledge representation and reasoning, leading to our proposed HPIPO framework.

To determine Host-Pathogen Interaction (HPI) mechanisms of infectious disease outcomes, we propose four fundamental HPI Postulates as guides (**Figure 1**):

1. HPI evolutionary dispositions: Hosts (pathogens) have evolutionarily selected for dispositions to react to pathogens (hosts) to achieve the best possible outcome for the host (pathogen). This is the root cause of HPI dynamics and explains associated outcomes.
2. HPI dynamic outcomes: Host (pathogen) outcomes are mechanistically determined by the dynamic HPIs at the molecular and cellular levels. The host (or pathogen) genetic and phenotypic profile (such as the direct and co-morbid susceptibility factors of the host) affect HPIs in ways relevant to disease outcomes. Different conditions such as age and sex may also affect the disease outcomes.
3. HPI roles: Molecular parts of hosts and pathogens are relevant to HPI outcomes. These host/pathogen molecules and their molecular interactions in the HPI network play specific roles in determining disease outcomes.
4. HPI checkpoints: Certain HPI components – which we call HPI checkpoints – are critical for the progression of HPI processes that result in HPI outcomes. Internal or external interruption of processes occurring at HPI checkpoints, such as by administering a drug or vaccine to a host, may change disease outcomes by altering manifested HPI networks. Rational drug and vaccine design targeting HPI checkpoint intervention may promote positive host outcomes. Molecules may participate in checkpoint stages across a range of dynamic HPI processes.

Postulate 1 characterizes the root cause of HPI dynamics on the part of both the host and the pathogen as a disposition that is selected for by evolution (23). Here, pathogens include cell-derived organisms (e.g., bacteria and parasites) as well as viruses. For example, viruses such as SARS-CoV-2 evolved to have the disposition to replicate through interactions with a host. Meanwhile, the host has an evolutionarily selected for disposition to destroy invading pathogens and prevent their replication, often through sophisticated mechanisms evolved to ward off infection. Nevertheless, host defense mechanisms sometimes fail. For example, the evasion or hijacking of the host immune response is crucial to the spread of SARS-CoV-2 (24). In such cases, host immune responses may occur too late, so that the viral spread is already out of control and the host immune response leads to host death (25, 26). However, the host also has the opportunity to develop a protective resistance against infection (27), illustrating a metaphorical tug-of-war or arms race between host and pathogen.

Postulate 2 states that the disease outcomes derive from dynamic host-pathogen interactions. The interactions between host and pathogen may generate a cascade of molecular interactions inside the host or pathogen, and the variations associated with various elements in the interactions may change the disease outcomes. For example, comorbidities (e.g., hypertension) may influence COVID-19 disease outcomes by affecting molecular and cellular HPI dynamics. An illustration of the postulate can be seen in the deployment of individual- or population-based public health measures that reduce infection or disease severity by influencing how frequently humans are exposed to the virus, resulting in different outcomes of the disease.

Given the complexity of the dynamic HPI-outcome mechanisms, the traditional approach of simply identifying the complex HPI networks, such as the network in **Figure 1**, is insufficient for analyses of mechanisms of diverse disease outcomes. As a novel strategy, Postulate 3 proposes that different components during the HPI dynamics have differential evolving roles in affecting the ways in which dynamic host-pathogen interactions contribute to disease outcomes. Each specific HPI initiates a dynamic interaction network involving both the host and the pathogen, and each interaction or element in the interaction has its role in the network. The HPI may change a cascade of molecular interactions inside the host or pathogen. Postulate 3 tells us that roles of different molecules and interactions in the network should be carefully studied to precisely investigate the mechanisms of different disease outcomes.

Inspired by the “immune checkpoint theory” (28) that guides cancer immunotherapy development, we postulate that interrupting molecular interactions at specific checkpoints – by targeted drug or treatment – could promote the immune response of a host or the pathogenicity of a pathogen. Checkpoint roles involve molecular interactions on the side of either host or pathogen and may be initiated naturally or by administered drugs or vaccines. Note that, as in the case of immune checkpoints in cancer immunotherapies, there is also evidence that checkpoints play an important role in promoting the host immune response to pathogens (29).

The four HPI Postulates are interlinked. Postulate 1 lays out how observed HPIs have emerged owing to evolutionary pressures. Postulate 2 describes the relationship between dynamic HPIs and outcomes. Postulate 3 emphasizes the importance of evolving roles of HPI components to HPI-outcome dynamics. Postulate 4 extends Postulate 3 by highlighting how disruption of HPI components may affect HPI-outcome networks. Overall, the four postulates provide a foundation for effectively studying HPI mechanisms.

## Illustration of the HPI postulates with host-coronavirus interaction

In this section, we illustrate how the four HPI postulates may aid in the understanding of the HPI mechanisms effecting different outcomes of COVID-19.

In accordance with Postulate 1, coronaviruses and hosts have their respective root evolutionary dispositions to achieve their best outcomes during the host-coronavirus interactions. On the pathogen side, coronaviruses have the evolutionary disposition to infect and replicate inside host cells and then spread to infect additional hosts. The viruses have evolved to efficiently use the host’s genetic material replication mechanism to replicate their own genetic materials. On the host side, the host has the evolutionary disposition to increase its ability to defend against the virus infection. The host mechanisms include innate and adaptive responses. Host innate responses include rapid cytokine production to combat virus infection. Host adaptive responses, including antibody and cell-mediated immune responses, take time to arise, but they can stimulate virus-specific immune responses. These innate and adaptive immune responses have developed over eons of evolution. As such, a comprehensive understanding of diseases like COVID-19 must take into account both the host and the pathogen.

**Figure 2:**
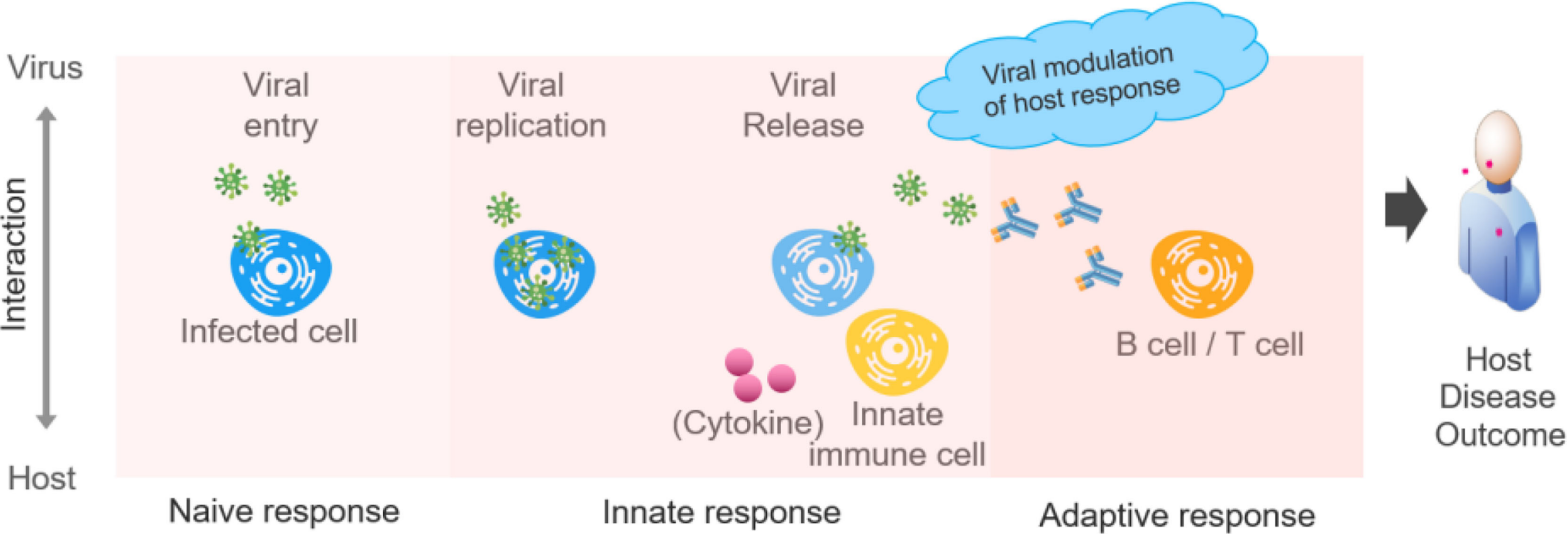
Model of host-coronavirus interactions and associated disease outcomes. The viruses enter, survive in, replicate in host cells, and may have genetic variations during the process. After initial naïve acceptance of viral entry without triggering an immune reaction (a naïve response), the host initiates active innate and adaptive responses. The host disease outcome will be determined by the dynamic host-coronavirus interactions.

As defined in the Postulate 2, the host (and pathogen) outcomes are mechanistically determined by, in this case, the dynamic host-coronavirus interactions at the molecular and cellular levels. This process is illustrated in **Figure 2**, which represents an integrative host-coronavirus interaction progression constructed on the basis of the HPI postulates. Progression includes four major viral processes and three major host processes. For coronaviruses, the four crucial processes are: (i) viral entry to host cell, (ii) viral replication, (iii) viral release from the infected cell, and (iv) modulation of host responses. Also – and sometimes dramatically – viral variants may be the result of host-virus interactions and viral reproduction (30, 31). For the host, the three responses include: (i) naïve immune response, (ii) innate immune response, and (iii) adaptive immune response. Each of these major processes includes many subprocesses, and disease outcomes can be explained by the degree to which host-pathogen interactions impact these subprocesses. If the virus “wins”, the result will be viral survival and replication; if the host “wins”, the host will eventually control viral replication and kill the viruses.

During the circulation and evolution of the virus in the host population, viral mutations, such as the D614G mutation in the spike (S) protein of SARS-CoV-2 (32), emerge to permutate their viral infectivity and pathogenicity, leading to different viral and host outcomes. Multiple genetic viral mutations may also co-exist and co-evolve with the host population (31). Our recent study of 6698 SARS-CoV-2 whole-genome sequences revealed four dominant mutations, including 5’UTR_c-241-t, NSP3_c-3037-t, NSP12_c-14408-t, and S_a-23403-g (33). These mutational changes in viral genomes all contribute to the rapid viral evolution and enhance viral transmission and long-term survival rates. In accordance with Postulate 2, these mutations change the host-coronavirus interactions at different stages. Also, as derived from Postulate 1, the viruses evolve to make various genetic mutations to achieve the best viral replication. Accordingly, the evolutions of the coronaviruses focus on increasing viral transmission, as demonstrated by the more transmissible Delta strain and then even more transmissible Omicron. Such a trend of increasing transmission supports increasing viral replications. Meanwhile, the pathogenesis of the SARS-CoV-2 in human hosts appears to vary, as shown by the more virulent Delta strain but less virulent Omicron compared to the original SARS-CoV-2 strain.

Host differences may result in different disease outcomes. For example, hosts exhibiting the angiotensin converting enzyme 1 (ACE-1) II genotype, which is more prevalent in Western Europe and steadily decreases in frequency towards Eastern Asia, appears to negatively correlate with the manifestation of COVID-19, and thus related mortality rates (34). Additionally, the C allele of SNP rs12242 in interferon-induced transmembrane protein 3 (IFITM3) was found to be associated with more severe COVID-19 disease in an age-dependent manner (35). Lastly, the COVID-19 severity is positively correlated with the hypertension comorbidity (36). If a patient already has hypertension, the further increased blood pressure due to the interactions would exacerbate the underlying comorbidity, which may explain the increased death rate of patients with superimposed COVID-19 and hypertension.

As described in Postulate 3, to better study the host-coronavirus interactions (HCI) and their relations with different disease outcomes, it is critical to identify the different roles of all HCI components. For example, the binding of the SARS-CoV-2 S protein to human angiotensin converting enzyme 2 (ACE2) leads to the viral invasion. Here the viral S protein plays the viral ligand role, and the human ACE2 protein plays the host receptor role. We can also go further to investigate the different parts of the S glycoprotein, which includes S1 and S2 regions. The S1 region also includes a receptor-binding domain that plays the specific role of binding to ACE2. As detailed later in this manuscript, based on prior knowledge of curated protein-protein interactions (PPIs) and domain-domain interactions (DDIs) (37, 38), we developed computational predictions based on host-coronavirus interactions, leading to the identification of potential effects of SARS-CoV-2 infection on different tissues and organs. Note that such potential effects may not be realized in all patients infected with SARS-CoV-2.

As another example illustrating Postulate 3, consider the differential roles of cytokines in COVID-19 disease progression. Although cytokines are known to play a critical positive role in defending against viral infection, they may also play negative roles. A cytokine storm is an excessive immune response to external stimuli associated with high levels of cytokine production (39). Cytokine storms are a major cause of acute respiratory distress syndrome and multiple-organ failure in severe COVID-19 patients, resulting in death within a short time (40). By identifying the different roles of these elements in the HCI-outcome networks, we can better understand the disease mechanisms under different conditions.

According to Postulate 4, some roles identified in the HPI processes can be defined as checkpoints critical to determining disease outcomes. For example, from the viral side, the SARS-CoV-2 S protein and its receptor-binding domain play a checkpoint role that facilitates viral invasion of host cells. Any mutation in the protein, esp. in its receptor-binding domain, may significantly affect the viral invasion and disease outcomes. Existing Pfizer-BioNTech and Moderna COVID-19 vaccines use the mRNA of the S protein for COVID-19 vaccine development. The COVID-19 vaccines can stimulate S protein-specific neutralizing antibody and thus block viral invasion. Similarly, therapeutical monoclonal neutralizing antibodies targeting the receptor binding domain of the S protein has also been found to substantially reduce the viral infection (41, 42).

From the host side, IL-6 is a checkpoint cytokine that is critical to regulating cytokine immune responses (39). Tocilizumab, a recombinant humanized anti-human IL-6 receptor monoclonal antibody, can bind to the IL-6 receptor with high affinity, and thus prevent IL-6 from binding to its native receptor and inducing downstream immune damage. It was recently reported that Tocilizumab improved the clinical outcome in severe and critical COVID-19 patients and effectively reduced mortality (43).

## Comparison: HPI postulates vs nearby postulates and theories

The HPI postulates are designed to aid in systematic modeling of HPIs and, in particular, illuminate why different people infected with the same pathogens have different outcomes. We emphasize that underlying HPIs at the molecular and cellular levels explain observed differential disease outcomes across hosts of the same pathogen. These postulates complement but differ significantly from existing postulates and theories as detailed below.

Koch’s postulates and its refinements (44–48) aim to confirm whether a pathogen is the cause of a disease. Specifically, Koch’s postulates require: (1) The microbe must be found in all organisms suffering from the disease but should not be found in healthy organisms. (2) The microbe must be isolated from a diseased organism and grown in pure culture. (3) The cultured microbe should cause the same disease when introduced into a healthy organism. (4) The microbe must be reisolated from the inoculated, diseased experimental host and be identical to the original causative agent. Koch’s postulates aimed to establish the causal relationships between exposure to a microbe and the emergence of a disease (44, 45, 47). Different from Koch’s postulates, our HPI postulates assume the microbial cause of a disease is known. Using technologies such as RT-PCR, we can now easily diagnose cases of COVID-19. The HPI postulates go further to determine why different outcomes result from the dynamic molecular and cellular interactions between the host and pathogen (**Figures 1**, **2**).

The Bradford Hill criteria aim to establish epidemiological evidence of causality between a presumed cause and an observed effect (49, 50). These criteria include a group of nine principles developed to establish epidemiological evidence of the causality between a presumed cause and an observed effect (49). In comparison, the identification of epidemiological evidence for such causality is not the focus of the HPI postulates. Instead, the HPI postulates are developed with the assumption that we already know the microbial cause of a disease. Importantly, the HPI postulates focus on the direct molecular and cellular host-pathogen interactions, rather than on the epidemiological evidence at the population level. There is, therefore, no conflict between these sets of frameworks and they serve to complement each other by addressing two different issues.

As was stated in the Introduction, the Damage-Response Framework (DRF) of microbial pathogenesis includes three tenets with the focus on understanding microbial pathogenesis based on the concepts of microbial pathogenesis, host damage, and microbial factors vs. the host response (2). Considering that the nature and mechanisms of microbial pathogenesis and damage are difficult to understand and characterize rigorously, we suggest the tenets of the DRF cannot serve as the most basic axioms or primitives for broad host-pathogen interaction studies. In contrast, our HPI postulates provide a more basic set of postulates and as such are a foundation that can, indeed, be used to explain the DRF.

## HPIPO: The hpi postulate and ontology framework applied to COVID-19

As illustrated in the embedded network shown in **Figure 1**, data and systems networks generated by *in vivo* and *in vitro* studies of complex host-pathogen interactions are often impressively large and detailed. Such networks include many nodes (entities) and edges (interactions), but the interaction types underlying each edge are usually unclear, and the roles played by the nodes and edges in the final disease outcomes are usually unknown. The data generated in conventional systems network representations are unintegrated, non-interoperable, and not machine-readable and thus form a hindrance to computer-assisted semantic analysis (51, 52). The pathways leading to specific disease outcomes and the measures to interrupt these pathways thus remain obscure.

To create more adequate representations of the vast stores of HPI data, we turn to ontologies. These are standardly built around an *is_a* relation linking one class of entities to its parent class. Ontologies are not, however, mere taxonomies; they provide additional relations between entities under different hierarchies. For example, an ontology might include a ‘*capable of* ’ relation, which links the class *‘Homo sapiens’* under the organism taxonomy hierarchy to the class *‘thinking’* in the Mental Functioning Ontology (53). Similarly, we can include a ‘*capable of binding to*’ relation to link the class ‘*spike glycoprotein (SARS-CoV-2)*’ to the class ‘*ACE2 angiotensin converting enzyme 2 (human)*’ in the Coronavirus Infectious Disease Ontology (CIDO) (19, 22).

To better represent HPI-outcome and pathogenesis mechanisms, we build on the HPI postulates to propose the Host-Pathogen Interaction Postulates and Ontology (HPIPO) framework, which uses the HPI postulates to guide the creation of ontological representations. HPIPO rests on the following three basic tenets:

- HPI postulates as the basis: The framework of the ontological representation of HPIs shall be rooted in the HPI postulates. Interoperable ontologies shall be used to semantically represent the entities and relations of specific molecular and cellular host-pathogen interactions and their associated participants, motivations, outcomes, roles, and checkpoints.
- Ontology usage: Consensus-based reference ontologies shall be used to logically represent HPIs and complex molecular and cellular interactions leading to various disease outcomes under specific conditions.
- Ontology interoperability: Terms, relations, and design patterns in the ontologies shall be aligned under the same interoperable ontology framework to achieve seamless data integration, sharing, and analysis.

Large-scale data interoperability is needed to apply the HPIPO framework and rely on the reuse of interoperable reference ontologies. Many well-curated ontologies covering nearby domains already exist, most notably in the Open Biomedical and Biological Ontologies (OBO) Foundry library, initiated in 2007 to support ontology interoperability through the adoption of a set of ontology development principles such as collaboration, openness and faithfulness to advances in scientific knowledge (54).

We envision that our proposed integrative HPIPO framework will serve as a foundation for ongoing and deeper studies of the COVID-19 disease. We initiated the development of the CIDO to serve as a logical framework for the systematic representation of the HCIs, disease outcomes, and the relations between the HCIs and disease outcomes (55). CIDO is interoperable with other ontologies such as the community-based Infectious Disease Ontology (IDO) (56) and Ontology of Host-Pathogen Interactions (OHPI) (38). The CIDO is an OBO library ontology that includes terminological content specific to coronaviruses, viral variations, hosts, phenotypes, host-coronavirus interactions, vaccines, drugs, and how these entities relate to each other (19, 22). In addition to asserting over 1,500 new terms, CIDO reuses over 10,000 terms from more than 30 other interoperable ontologies all aligned under the Basic Formal Ontology (BFO) (57), an upper-level ontology that is an International Organization for Standardization standard (ISO/IEC 21838-2:2021). As BFO has been widely used in over 450 ontologies, its adoption allows us to align imported and new terminological content under a unified ontology framework.

**Figure 3:**
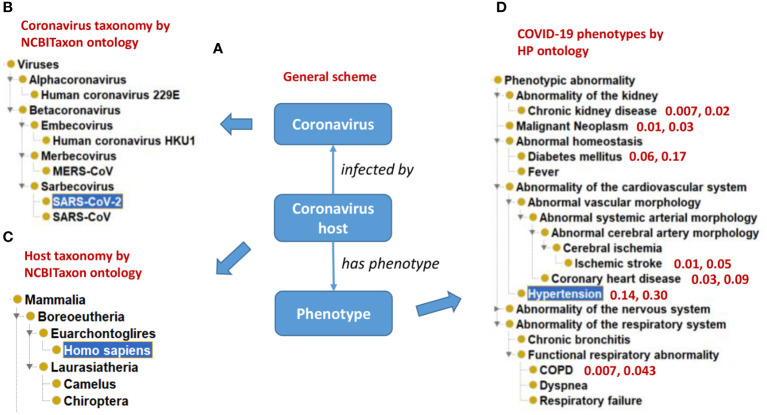
Ontological representation of coronaviruses, hosts, and phenotype outcomes of host-coronavirus interactions. **(A)** A general scheme. **(B)** The NCBITaxon taxonomical hierarchy of representative human coronaviruses. **(C)** The taxonomical hierarchy of representative hosts of coronaviruses. **(D)** Human Phenotype Ontology (HPO) hierarchy of representative human phenotypes commonly seen in COVID-19 patients. Representative comorbidity phenotypes and associated phenotype frequency in mild and severe COVID-19 patients are also represented. For example, “0.14, 0.30” in **(D)** indicates that superimposed hypertension is found in 14% of mild symptom patients and 30% of severe symptom patients. The results were summarized from reported literature (58, 59).

**Figure 3** illustrates how CIDO imports terms from many existing reference ontologies to represent coronaviral knowledge from different aspects. Specifically, the taxonomic hierarchies of various coronaviruses and their hosts are extracted from the NCBI Taxonomy ontology (NCBITaxon) (60) (**Figures 3A-C**), and the representative COVID-19 symptoms are represented using Human Phenotype Ontology (HPO) (61) terms (**Figure 3D**). Our ontology-based analysis also found that those patients with many comorbid conditions (e.g., chronic kidney disease, hypertension, and diabetes) are at heightened risk for severe symptoms and death (36) (**Figure 3D**).

CIDO defines new axioms to link different kinds of entities (19, 22). For example, the process ‘*SARS-CoV-2 S-ACE2 binding*’ is defined in CIDO by three logical axioms expressed in the Web Ontology Language (OWL) (expressions in **bold** are logical connectives; expressions in *italics* are classes or relations):

*has participant* **some** *SARS-CoV-2 S protein* **and** *(has role* ***some*** *ligand role)*
*has participant* **some** *ACE2* **and** *(has role* ***some*** *receptor role)*
*activated by* **some** *TMPRSS2* **and** *(has role* ***some*** *enzyme activator role)*

The above axioms define logical relations obtained when a SARS-CoV-2 S protein-human ACE2 binding process has two participants: a SARS-CoV-2 S protein that serves as a ligand role, and a human Angiotensin-Converting Enzyme 2 (ACE2) protein that serves as a receptor role. This binding is activated by TMPRSS2, a type 2 transmembrane serine protease 2 that activates the binding between S protein and ACE2 by priming the S protein through S protein cleavage, allowing the fusion of the viral and cellular membranes (62). This binding process provides the molecular mechanism for the coronavirus to invade host cells.

**Figure 4:**
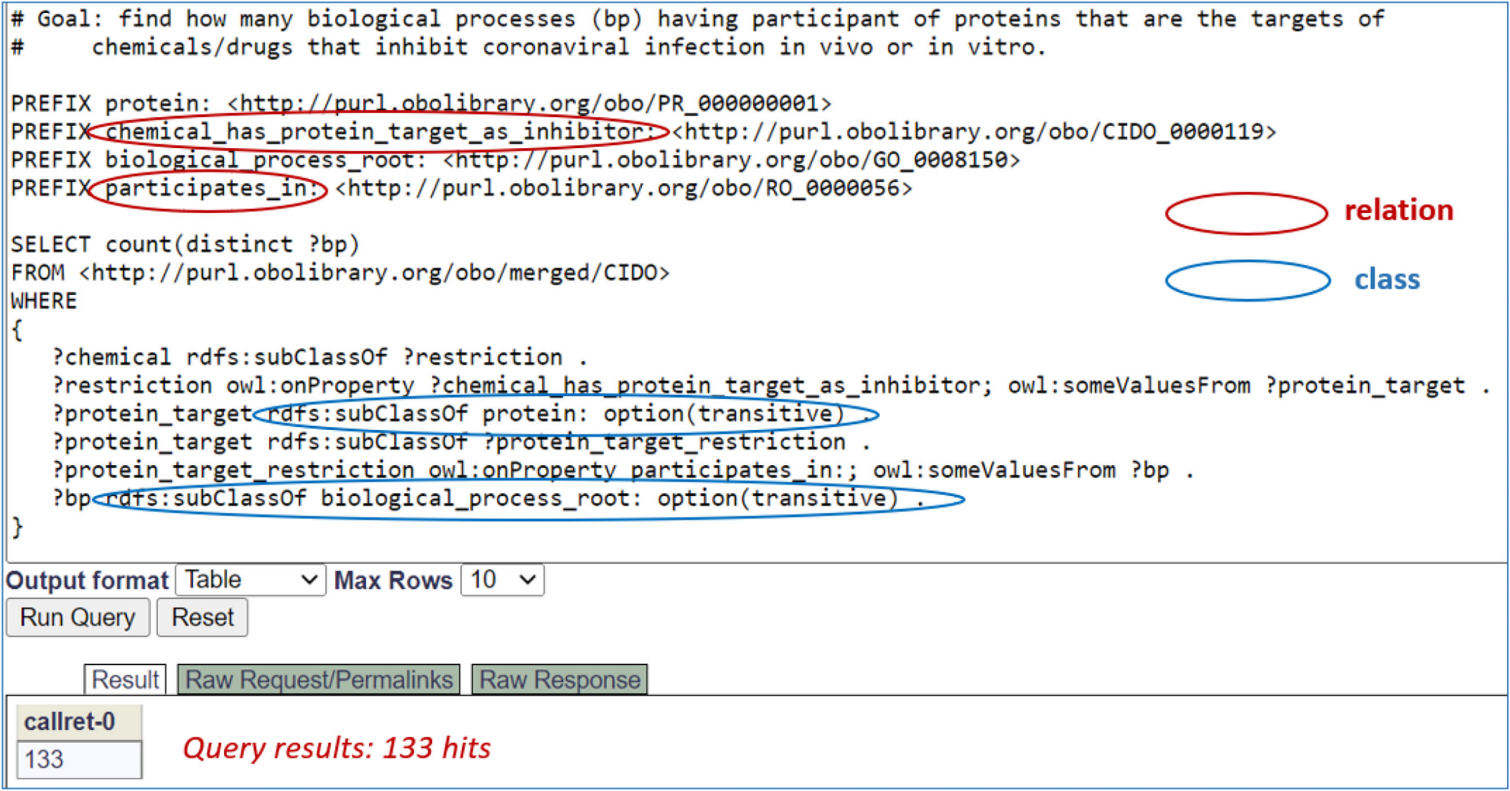
SPARQL query of CIDO for anticoronaviral chemicals/drugs out of host-coronavirus interactions. This SPARQL identified 125 biological processes having participant of proteins that are the targets of chemicals/drugs that inhibit coronaviral infection *in vivo* or *in vitro*, illustrating how the relations (red circles) and classes (blue circles) are associated and interlinked. The SPARQL was performed using Ontobee SPARQL endpoint (http://www.ontobee.org/sparql). The detailed information about these 125 biological processes and their associated proteins and chemicals/drugs is provided in **Supplemental Figures 1, 2**.

Using semantic axioms as illustrated above, we can query the CIDO ontology to extract various types of knowledge. For example, we can recursively query the CIDO triple store using SPARQL query scripts (63) to find those drugs and biological processes where the drugs are capable of interrupting specific protein targets participating in specific biological processes in host-coronavirus interactions. **Figure 4** presents CIDO-based SPARQL query results identifying 133 biological processes, such as ‘*positive regulation of vasoconstriction’* (GO_0045907), ‘*response to glucocorticoid*’ (GO_0051384), and ‘*peptidyl-tyrosine phosphorylation*’ (GO_0018108), which involve proteins as the inhibitory targets of chemicals/drugs that inhibit coronaviral infection *in vivo* or *in vitro*. **Supplemental Figure 1** and **Supplemental File 2** provide more details about the identified 133 processes, 125 proteins that participate in these processes, and 52 chemicals/drugs that play the inhibitor role.

Using the postulates, we can identify and link specific roles and checkpoints to specific entities in the HPI interactions leading to disease outcomes. We can also use this ontological representation to interpret conventional systems networks such as that embedded in **Figure 1**, making these networks amenable to computer-assisted reasoning. With new results reported, we could use the eXtensible ontology development (XOD) principles and related tools (52) to recursively update CIDO as illustrated in our recent study (64).

Lastly, it is worth noting that the ontology data representation model in the HPIPO framework is a departure from, but improvement on, the traditional use of ontologies for annotation. Gene products, for example, are frequently annotated with terms from GO (65), resulting in interoperable annotations of components in molecular interactions. Guided by the HPI postulates, the CIDO ontology goes further by representing how coronaviruses infect host organisms, induce specific molecular interactions, and thereby cause different disease outcomes. CIDO thus provides a direct ontological representation of specific interactions. CIDO modeling shares similarities with the GO Causal Activity Modeling (GO-CAM) (66), which provides a framework for representing qualitative causal models for how gene products act together to conduct a biological program. With CIDO, however, modeling aims to represent ontologically the whole HPI cycle from pathogen infection to disease outcomes.

In each respect, we intend the HPIPO framework to either complement or be compatible with existing nearby approaches to investigating disease outcomes. As should be clear, however, HPIPO differs in significant ways from nearby proposals.

## Applications: Rational COVID-19 Cocktail Design and Granular PPI Analysis

In this section, we first demonstrate how the HPI postulates and their ontological representation (HPIPO) can be used to support rational drug and vaccine design. Given the complex host-COVID-19 interactions, we propose a drug cocktail strategy and a vaccine cocktail strategy for enhanced drug/vaccine design, and ontology modeling can help such rational design. Following this application – and building on previous work involving human-coronavirus protein-protein interaction network analyses – we identify how the HPIPO framework can aid researchers in highlighting important mechanisms within a PPI interaction network.

No single drug has been proven to be exceedingly effective against COVID-19. Remdesivir (Veklury) is the first FDA-approved antiviral drug to treat COVID-19 in patients who are aged 12 or older. Remdesivir interferes with the action of viral RNA-dependent RNA polymerase (RdRp), decreasing the viral RNA production. Paxlovid and molnupiravir are two more drugs authorized by FDA for emergency use (67). Paxlovid includes two active ingredients: nirmatrelvir and ritonavir. Nirmatrelvir blocks Mpro, an enzyme needed for SARS-CoV-2 virus to replicate. Ritonavir helps slow the breakdown of nirmatrelvir in the body. Molnupiravir is the isopropyl ester prodrug of the ribonucleoside analogue β-D-N4-hydroxycytidine (NHC), which inhibits viral reproduction by promoting mutations in the viral RNA replication by RdRp. Several monoclonal antibodies, such as bebtelovimab and sotrovimab that target the spike protein of SARS-CoV2, have also been authorized for emergent usage against COVID-19. Each of these drugs is partially effective, and it is highly desired to develop a treatment recipe to fully treat the disease.

Aligned with the HPIPO, we have previously proposed a related “Host-Coronavirus Interaction (HCI) checkpoint drug cocktail” strategy (22). Basic to this strategy is the thesis that drugs can be developed to interrupt specific ‘checkpoints’ and so block the emergence of severe disease outcomes. Since HCIs often result in distinct outcomes under similar conditions, and since no single drug intervention has yet had dramatically positive effects in the case of COVID-19, we hypothesize that a drug cocktail to block the formation of specific outcomes will be more effective. The individual drugs in such a cocktail would interrupt specific HCI pathways at different disease stages, and thereby lead to more favorable outcomes. Such a cocktail strategy is also inspired by HIV research. To treat HIV patients (68, 69), the combination drug treatment known as the highly active antiretroviral therapy (HAART), or simply “AIDS cocktail”, was initiated in 1995, and since then has made AIDS a manageable disease.

**Figure 5:**
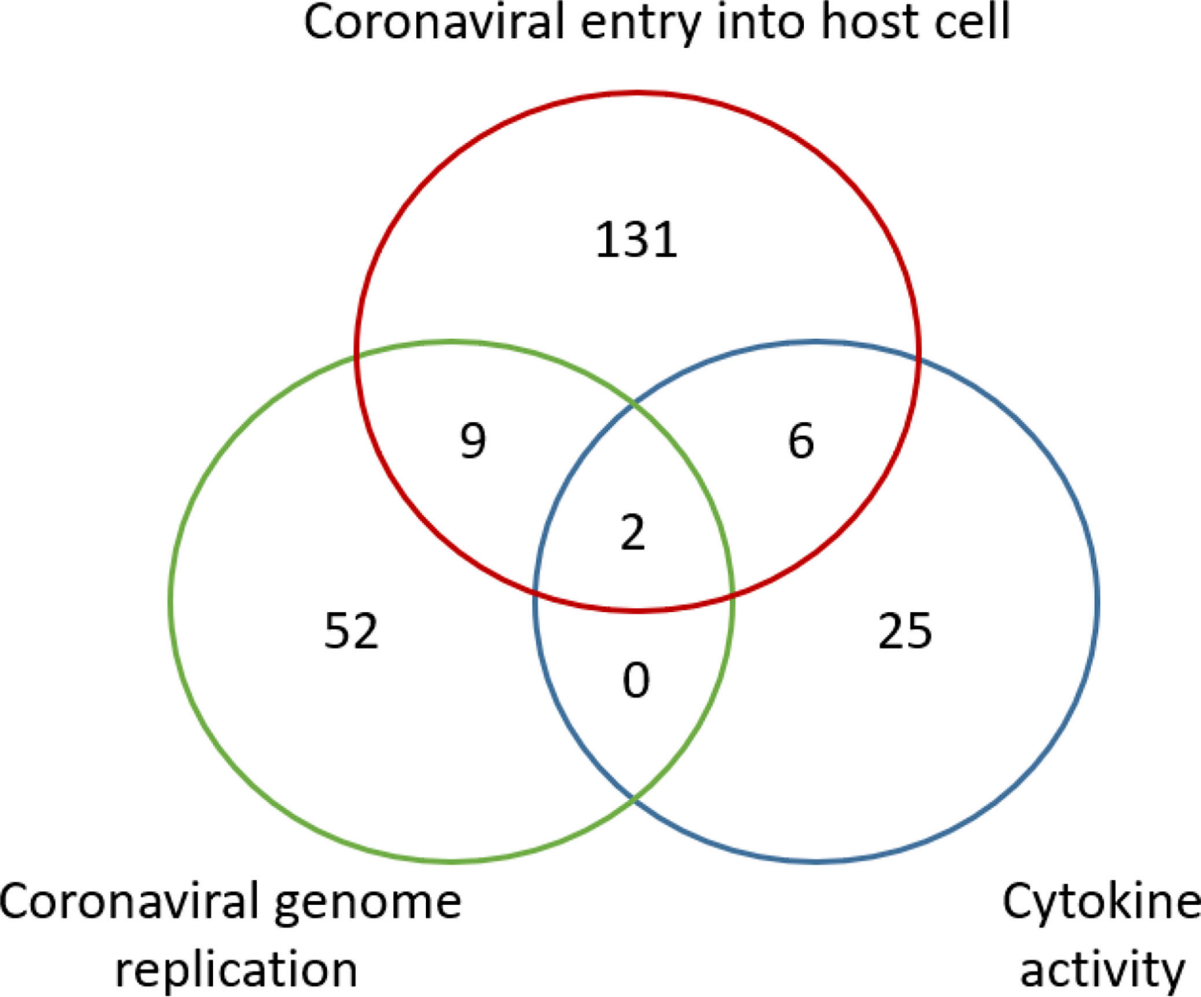
Venn Diagram of potential COVID-19 drugs based on the HPI Postulate drug cocktail strategy. A total of 232 drugs were identified to have their protein targets involving coronavirus entry, coronavirus genome replication, and host cytokine activity against COVID-19. Two drugs (i.e., copper and artenimol) were shared to have protein targets involved in all three processes. The drug screening study was performed using the DrugXplore program (70).

Based on the cocktail drug design strategy, it would be reasonable to repurpose drugs that might interrupt important checkpoints in the dynamic HCI network. Based on this strategy, we have developed a cocktail drug screening algorithm in the DrugXplore program (70). Using this algorithm, we identified a total of 232 drugs that have their drug protein targets involving coronavirus entry, coronavirus genome replication, and host cytokine activity against COVID-19 (**Figure 5**). Two drugs (i.e., copper and artenimol) were also found to have protein targets involved in all the three processes. Although individual cocktail drugs do not have to target multiple HCI processes simultaneously, this study provides a proof-of-concept demonstration on how to develop cocktail drugs against COVID-19.

For successful cocktail drug design, we will require systematic semantic understanding of HCI-outcome pathways, individual interactions in the pathways, and the roles of the components in the interactions and pathways. To achieve this, the HPIPO framework applied to host-coronavirus interactions is advantageous as it aids the representation of HCI-outcome pathways, highlighting important steps in these complex processes. Moreover, our previous studies (15, 22, 36, 71) provide a demonstration of how the CIDO can be used to semantically represent various HCIs, HCI-associated entities, and drugs that interrupt specific entities in the HCIs. Such representation provides the foundations needed for the development of tools supporting intelligent queries (22, 72, 73) and enhanced computational predictions (11, 74, 75) from ontology-represented data. For example, using the semantic patterns illustrated by axioms defined in the ontology, we were able to recursively query the ontology CIDO itself to find those drugs and biological processes where the drugs (e.g., amodiaquine (76), dasatinib (77), imatinib (78), nilotinib (79), and ouabain (80) are capable of interrupting specific protein targets participating in specific biological processes in HCIs (22).

HPIPO provides a logical and effective framework to further develop the CIDO to include the large volume of new findings in HPI-outcome and drug responses. It is also noted that not all the HPI relationships identified in experimental and clinical studies will reflect the causal mechanisms of disease outcomes. Many show just the correlation between these outcomes and the infection by a microbe. Since the HPI postulates emphasize the annotation of roles of different components in the HPI-outcome processes, the HPIPO framework provides a feasible path toward tackling such issues.

Meanwhile, we have also used reverse vaccinology and machine learning strategy to predict protective antigen candidates for COVID-19 vaccine development (71). Our study independently predicted the S-protein as the top-ranked protective antigen. Furthermore, we predicted that nonstructural proteins (Nsps) Nsp3 and Nsp8, are also effective protective antigens, and we found that nonstructural proteins Nsp3, 3CL-pro and Nsp8 are adhesins critical for viral adherence and invasion (71). Nsp3 is a key component of the viral replication. We further demonstrated that Nsp3 can stimulate both MHC class I and II epitopes as well as B-cell epitopes (71). In addition, there are experimental studies that confirm our computational predictions. For example, the papain-like protease PLpro, a sub-domain of Nsp3, regulates SARS-CoV-2 viral spread and innate immunity (81), and Nsp3 induces both strong CD4+ helper T cells and CD8+ cytotoxic T-cell responses (82). Based on these results, we proposed to develop a “Sp/Nsp cocktail vaccine”, which would include both the structural S protein and either nonstructural protein Nsp3 or Nsp8. Such a cocktail is likely to induce a stronger and more sustained cell-mediated and humoral immunity necessary to prevent viral invasion and replication, avoid immune evasion, and control the viral infection (71). Such a cocktail vaccine strategy is also aligned and can be better studied with the HPIPO framework.

Furthermore, it is possible to combine the “Host-Coronavirus Interaction (HCI) checkpoint drug cocktail” strategy and the “Sp/Nsp cocktail vaccine” strategy to form a complementary cocktail drug/vaccine strategy in our systematic design against the highly transmissible and deadly COVID-19 disease. The cocktail vaccine would stimulate both humoral and cell-mediated host immune responses against viral invasion and vial immune evasion. The vaccine-targeted viral targets should be specific checkpoints of the host-coronavirus interaction (HCI) network. The drug cocktail would further treat the patients at the critical checkpoints of the HCI network. Both vaccine- and drug-targeted HCI checkpoints may be considered simultaneously. Our HPIPO framework will help us to identify roles and checkpoints for different components in the HCI-outcome analysis. The CIDO ontology can be leveraged to semantically represent the large amounts of knowledge and data. Computational machine learning methods can also be further developed. New hypotheses will also be generated for experimental evaluations.

**Figure 6:**
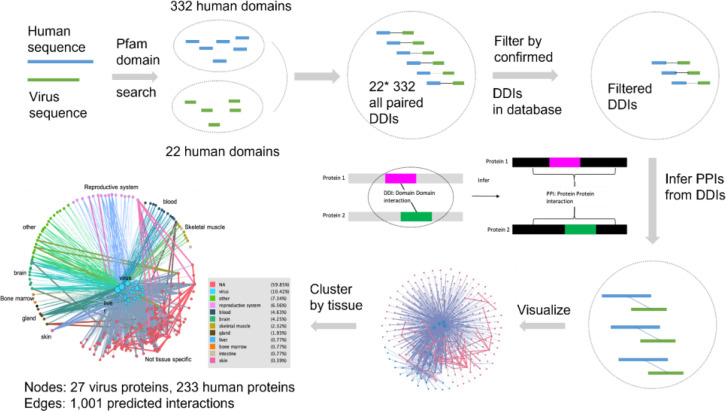
Predicted human-coronavirus protein-protein interactions (PPIs) and their affected tissues. The predicted 1,001 interactions that involve 27 virus proteins and 233 human proteins are detailed in **Supplemental File 3**. The predicted tissue specificity results are provided in the **Supplemental File 4**. See the text for more details.

In addition to facilitating the creation of vaccines and drug cocktail, the HPIPO framework can be used to help predict how a set of proteins might interact. **Figure 6** represents computational predictions of human-coronavirus protein-protein interactions (PPIs) based on prior knowledge of curated PPIs, domain-domain interactions (DDIs) (37, 38) and sequence similarity (83–86). Focusing on DDIs, we were in particular able to infer potential interactions between proteins if one domain in a pathogen protein A interacted with another domain in a host protein B. For example, when applying this strategy to analyze human sequences and virus sequences, we identified 332 human domain and 22 virus domain pairings. After filtering using prior knowledge of confirmed DDIs, we were able to predict 1,001 interactions that involve 27 virus proteins and 233 human proteins (See **Supplemental File 3**). The interacting human proteins are annotated with tissue-specific expression profiles from the Human Protein Atlas (37, 87), downloaded at 2020-4-18. In the last image of **Figure 6**, nodes in the middle are virus proteins, nodes in the outer circle are human proteins, and proteins enriched in different tissues are marked with different colors. The tissue specificity results are provided in the **Supplemental File 4**.

**Figure 7:**
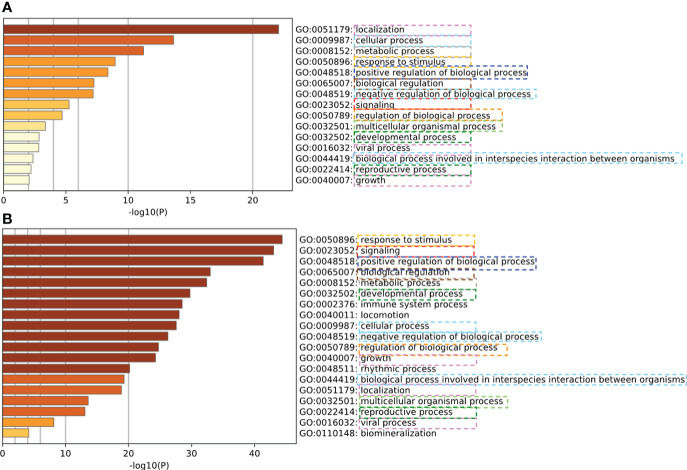
Verification of predicted human-coronavirus protein-protein interactions (PPIs) with experimentally validated PPIs. **(A)** Enriched GO terms of experimentally validated PPIs. **(B)** Enriched GO terms of our predicted PPIs. Our predicted PPIs (**Figure 6**) have coherent informative GO term annotations with the experimentally validated PPIs.

To verify our predicted PPIs, a computational assessment (88) was implemented. Specifically, semantically enriched Gene Ontology (GO) (65) terms representing host proteins predicted to be targeted by pathogens were used to evaluate the functional relevance of predicted host-pathogen PPIs (89). We evaluated the consistency of the enriched GO terms of experimental validated PPIs and our predicted PPIs. The experimental PPIs were collected from 332 high-confidence protein-protein interactions between SARS-CoV-2 and human proteins which were identified by affinity-purification mass spectrometry (90). As shown in **Figure 7**, the enriched GO terms from experimental PPIs and our PPI largely overlapped, which provides strong evidence that our prediction is accurate and meaningful.

Important for our purposes is that the complexity of the domain of DDI research tends to result in protein domain interactions represented in large, unwieldy, webs of nodes and edges which are difficult to parse and challenging to analyze. As we have illustrated, ontologies are useful tools for highlighting important connections exhibited by complex domains. DDI research is not presently represented in any semantically interoperable ontology. Standardizing existing DDI knowledge in an ontology will, we suggest, provide a firm foundation on which to add novel DDI knowledge, and so potentially provide more accurate and reliable predictions of important PPIs. New DDI-based human-pathogen PPI prediction methods can be developed to overcome many issues such as domain sequence variations and the huge differences in the interaction interface (85). It is also possible to ontologically model and represent the sequence variations and interaction interfaces, leading to more advanced PPI predictions.

Formally representing PPI knowledge would, moreover, bring such research within the scope of our HPIPO framework. We have, for example, emphasized the importance of molecular roles in disease outcomes, but our postulates may apply equally at the level of protein domains, as well as constituent proteins. Put another way, protein domains too might be said to bear HPI roles, acquired from the relevant molecular parts of hosts and pathogens as described in Postulate 3.

## Discussion and future work

Three major contributions are made in this study. First, we proposed four HPI postulates as the basis for thorough and systematic understanding of the molecular mechanisms of diverse disease outcomes. Second, we proposed the HPIPO framework to semantically model, represent, and analyze the HPI details derived from the application of the HPI Postulates. Third, we have applied the HPIPO framework to study the COVID-19 disease and the host-coronavirus interactions, and further proposed a complementary drug and vaccine cocktail strategy against COVID-19, while motivating potential utility of characterizing PPI research within the scope of the HPIPO framework.

The driving scientific question motivating our HPI postulates and HPIPO framework concerns why people infected with the same SARS-CoV-2 virus may manifest different symptoms and disease outcomes. This is not addressable by Koch’s Postulates, which aim to build up a set of criteria to establish whether a specific organism is the cause of a specific disease. The Damage Response Framework (DRF) (2) emphasizes the amount of damage caused by microorganism to the host and its role in the host-relevant outcome, but it cannot evaluate *how* or *why* a pathogen causes the amount of damage that it does cause from a granular perspective of HPI mechanisms. In comparison, our HPI postulates address the issue of deep understanding of specific molecular HPI mechanisms. It is possible to use the HPI postulates to explain the microbial pathogenesis and damage, further supporting the DRF applications.

The four HPI postulates provide rich details of HPI mechanisms. First, the postulate of HPI evolutionary dispositions addresses the issue of the root cause of the pathogen and host behaviors in the HPI. The postulate of HPI dynamic outcomes lays out the HPI dynamic processes leading to specific disease outcomes. Various conditions (e.g., biological sex and age) in the HPI dynamics may affect the disease outcomes. Furthermore, we propose two novel postulates of HPI roles and HPI checkpoints. The postulate of HPI roles explains the HPI-to-outcome dynamics by assuming that different HPI components have specific roles. Furthermore, the postulate of HPI checkpoints assume that some HPI components have specific checkpoint roles, which are critical roles to the realization of critical disease outcomes. To undercover the fundamental HPI-to-outcome mechanisms, it is important to identify those checkpoint molecules. Like the inhibition of immune checkpoint molecules leading to cancer immunotherapy (28), the HPI checkpoint molecules can also be interrupted for translational prevention or therapy purpose.

While the set of four HPI postulates explain the molecular HPI-to-outcome mechanisms well, a specific strategy is needed to fully apply the postulates for deep HPI studies. This is why we propose the HPIPO framework, in which interoperable ontologies are used to implement the HPI postulates, in the interest of supporting standardized knowledge representation, data annotation and sharing, and computer-assisted data analysis. It is also noted that as the core of the HPIPO framework, the HPI postulates can exist independently and be used for other purposes such as the explanation of different HPI-to-outcome phenomena. It is feasible to use the HPI postulates to explain the Damage Response Framework (DRF). It is also likely that we can apply the HPI postulates for developing ontology-independent technical strategies.

The HPIPO framework is illustrated by focusing on human-coronavirus interactions in this manuscript. In such interactions, the hosts and patients have different evolutionary dispositions, which form the basis of the interactive human and coronavirus response profiles. Analyses of relevant underlying molecular and cellular HPIs frequently result in extensive, difficult to analyze, networks of interactions from both the host and coronavirus sides. To better understand these extensive networks and interactions, using the HPIPO framework we emphasize roles of molecules in specific pathways of coronaviral pathogenesis. Moreover, our application of the HPIPO framework reveals the importance of certain checkpoints in such pathways, where importance stems from the fact that had these checkpoints been different, the pathway itself would have been substantially altered. Just as we see in cancer immune checkpoint theory applications, identification of such checkpoint molecules will allow more effective rational COVID-19 drug design, therapeutic treatments, and preventative measures.

As noted, PPI networks, such as that represented in **Figure 1** and found in various systems network studies (91–93), are often challenging to integrate and use in an automated way. Our proposed HPIPO framework provides a novel approach to such research, and one which will overcome interoperability challenges. PPI network entities represented in well-designed, curated, ontologies such as those extending from BFO, would already be interoperable with a wide range of biomedical and biological ontologies and associated data. Once standard PPI network entities and relations have been represented in such ontologies, the results of new network research could be connected – using computational methods – to the results of existing network research represented in ontologies.

Computational predictions of HPIs at the proteome, genome, and epigenome level (94–96) have accumulated large amounts of prior knowledge and principles. However, it is often unclear how useful the identification of large numbers of host-pathogen PPIs might be to researchers due to many technical issues (95). Various factors may affect the prediction outcomes. Our study (**Figures 6**, **7**) showed that DDIs can be further studied and used for PPI analysis. However, factors such as domain sequence variations, interaction interface differences, promoters and transcription factors (97), and general host environmental conditions may also influence PPIs and disease outcomes. Furthermore, experimental results may also vary in apparently similar experimental designs (98–100). These phenomena indeed align with our postulate of HPI dynamic outcomes in that various conditions may affect the HPI dynamics and disease outcome. To address these issues, it is possible to develop and follow minimal information standards (9, 101), dissect different conditions and variables in specific experimental and computational studies, and model and represent these conditions and variables using interoperable ontologies (9, 51, 98, 99, 102–104). Ontology-based knowledge bases (38, 100, 105) can also be used and applied. The usage of these standards and ontologies will significantly improve our reproducible and interoperable HPI studies, and help develop more advanced prediction methods.

There are many other possible applications of the HPI postulates and HPIPO framework, such as investigation into novel emerging diseases and associated disease pathways. Identifying the molecular constitution of a novel pathogen is an important first step in combatting future infections. We must also understand how novel emerging pathogens spread, as well as the underlying mechanisms that result in observed disease outcomes. Our strategy emphasizes curating and leveraging known molecular information relevant to pathogens and hosts in the interest of identifying checkpoints pivotal to observed disease outcomes. We wager that such careful curation will result in much easier and efficient strategies for predicting disease outcomes and designing drugs, treatments, and vaccines.

There is, of course, much more work to be done in developing, refining, and applying the HPIPO framework. We would like to ask the ontology and HPI communities to aid in our efforts in applying and refining the HPIPO framework. Areas in need of further development include: the annotations of roles relevant to HPI outcomes in conventional network analyses, implementation of the HPIPO framework in data analyses and software development, as well as continued term development in CIDO (19) following OBO Foundry principles (54).

## Data availability statement

The original contributions presented in the study are included in the article/**Supplementary Material**, further inquiries can be directed to the corresponding authors.

## Author contributions

HY and YW: COVID-19 domain expert and CIDO development. LL, TZ, JZ, PL, Z-PL, and LChen: Protein-Protein Interaction (PPI) analysis and prediction. AH: CIDO development and data analysis. JB, EM, and BS: Evaluation of CIDO ontology, postulates, and frameworks. JH and H-HH: Host-coronavirus interaction (HCI) knowledge mining, postulate evaluation, and result interpretation. YL and ZW: COVID-19 drug analysis. EO and LCheng: Statistical data analysis expert. XZ, XY: COVID-19 clinical domain experts. SH, JS, KE, GH, GO, and BA: Biomedical domain experts, postulation evaluation and result interpretation. YH: Project design, initiation of proposed HPI postulates and HPIPO framework, microbiology expert, and CIDO and SPARQL script development. YH, HY, LL, AH, and JH drafted the first manuscript draft. All authors contributed to the article and approved the submitted version.
