# Supplementary material for: A new framework for host-pathogen interaction research
Source: Front Immunol. 2022 Dec 15;13:1066733. doi: 10.3389/fimmu.2022.1066733 (PMC9797517; doi:10.3389/fimmu.2022.1066733)
Supplement: Supplementary file 1 [file DataSheet_1.zip › New folder (2)/Supplemental File 1.PDF]

Supplemental File 1. SPARQL queries of CIDO ontology

Instruction: The following SPARQL scripts can be executed by copying the code to Ontobee SPARQL endpoint site and run:
https://ontobee.org/sparql

Two SPARQL scripts are provided below.

(1) SPARQL script for Fig. 4.

```
--
# Date: 10/13/2020 -- by Oiver He
#Goal: find how many biological processes (bp) having participant of proteins that are the targets of
#     chemicals/drugs that inhibit coronaviral infection in vivo or in vitro.

PREFIX protein: <http://purl.obolibrary.org/obo/PR_000000001>
PREFIX chemical_has_protein_target_as_inhibitor: <http://purl.obolibrary.org/obo/CIDO_0000119>
PREFIX biological_process_root: <http://purl.obolibrary.org/obo/GO_0008150>
PREFIX participates_in: <http://purl.obolibrary.org/obo/RO_0000056>

SELECT count(distinct ?bp)
FROM <http://purl.obolibrary.org/obo/merged/CIDO>
WHERE
{
   ?chemical rdfs:subClassOf ?restriction .
   ?restriction owl:onProperty ?chemical_has_protein_target_as_inhibitor;
owl:someValuesFrom ?protein_target .
   ?protein_target rdfs:subClassOf protein: option(transitive) .
   ?protein_target rdfs:subClassOf ?protein_target_restriction .
   ?protein_target_restriction owl:onProperty participates_in:; owl:someValuesFrom ?bp .
   ?bp rdfs:subClassOf biological_process_root: option(transitive) .
}
--
```

Note:
See more explanation about how to run SPARQL here in our Ontobee SPARQL tutorial:
https://ontobee.org/tutorial/sparql

(2) SPARQL script that provides details related to the for Fig. 4.

```
# Date: 11/8/2022 -- by Oiver He
# Goal: find the details of the biological processes (bp) having participant of proteins that are the targets
#  of  chemicals/drugs that inhibit coronaviral infection in vivo or in vitro. Note that different from the
#  above script, this SPARQL script provides detials of the biological processes, chemicals, and proteins.

PREFIX protein: <http://purl.obolibrary.org/obo/PR_000000001>
PREFIX chemical_has_protein_target_as_inhibitor: <http://purl.obolibrary.org/obo/CIDO_0000119>
```

```
PREFIX biological_process_root: <http://purl.obolibrary.org/obo/GO_0008150>
PREFIX participates_in: <http://purl.obolibrary.org/obo/RO_0000056>

SELECT distinct ?bp ?bp_label ?chemical STR(?chemical_label) as ?chemical_label
        ?protein_target STR(?protein_target_label) as ?protein_target_label
FROM <http://purl.obolibrary.org/obo/merged/CIDO>
WHERE
{
  ?chemical rdfs:subClassOf ?restriction .
   FILTER regex(str(?chemical),'CHEBI','i') .
  ?restriction owl:onProperty ?chemical_has_protein_target_as_inhibitor;
owl:someValuesFrom ?protein_target .
  ?protein_target rdfs:subClassOf protein: option(transitive) .
  ?protein_target rdfs:subClassOf ?protein_target_restriction .
  ?protein_target_restriction owl:onProperty participates_in:; owl:someValuesFrom ?bp .
  ?bp rdfs:subClassOf biological_process_root: option(transitive) .

  ?bp rdfs:label ?bp_label .
  ?chemical rdfs:label ?chemical_label .
  ?protein_target rdfs:label ?protein_target_label .
}
```

--

Note:

Supplemental File 2 provides the results of the above SPARQL script execution in http://sparql.hegroup.org/sparql/ .

Also noted, more CIDO related SPARQL scripts are available here on CIDO GitHub website: https://github.com/CIDO-ontology/cido/blob/master/docs/sparql.txt
