# Supplementary material for: A new framework for host-pathogen interaction research
Source: Front Immunol. 2022 Dec 15;13:1066733. doi: 10.3389/fimmu.2022.1066733 (PMC9797517; doi:10.3389/fimmu.2022.1066733)
Supplement: Supplementary file 1 [file DataSheet_1.zip › New folder (2)/Supplemental File 3.PDF]

Virun_protein,Human_protein
YP_009725303.1,Q9BQ66
YP_009725303.1,P21246
YP_009725318.1,P21246
YP_009725299.1,P27986
YP_009725299.1,P46108
YP_009725299.1,P35869
YP_009725299.1,P06400
YP_009725299.1,P49841
YP_009725299.1,P55211
YP_009725299.1,Q12933
YP_009725299.1,P51693
YP_009725299.1,P05067
YP_009725299.1,P25445
YP_009725299.1,P63165
YP_009725299.1,Q13158
YP_009725299.1,Q14790
YP_009725299.1,P61586
YP_009725299.1,P60953
YP_009725299.1,P04637
YP_009725299.1,Q12959
YP_009725299.1,Q07817
YP_009725299.1,P10415
YP_009725299.1,P55957
YP_009725299.1,P24385
YP_009725299.1,P31946
YP_009725299.1,P22681
YP_009725299.1,P00533
YP_009725299.1,P62993
YP_009725299.1,P20248
YP_009725299.1,P24941
YP_009725299.1,P11802
YP_009725299.1,P30281
YP_009725299.1,Q9Y4K3
YP_009725299.1,P06493
YP_009725299.1,P42768
YP_009725299.1,P38936
YP_009725299.1,Q8N726
YP_009725299.1,P17676
YP_009725299.1,Q13485
YP_009725299.1,P62826
YP_009725299.1,P25963
YP_009725299.1,Q92793
YP_009725299.1,P46109
YP_009725299.1,P67870
YP_009725299.1,P35222
YP_009725299.1,P63279
YP_009725299.1,Q01094
YP_009725299.1,P08047
YP_009725299.1,P26641
YP_009725299.1,P29353
YP_009725299.1,P40763
YP_009725299.1,P04626
YP_009725299.1,Q13541

YP_009725299.1,Q09472
YP_009725299.1,Q04206
YP_009725299.1,P62942
YP_009725299.1,P12956
YP_009725299.1,Q06124
YP_009725299.1,P20226
YP_009725299.1,Q13547
YP_009725299.1,Q92769
YP_009725299.1,P01112
YP_009725299.1,P03372
YP_009725299.1,P63244
YP_009725299.1,Q99836
YP_009725299.1,P05106
YP_009725299.1,Q15628
YP_009725299.1,P06239
YP_009725299.1,Q15796
YP_009725299.1,P84022
YP_009725299.1,Q00987
YP_009725299.1,P01106
YP_009725299.1,P16333
YP_009725299.1,P12004
YP_009725299.1,P28340
YP_009725299.1,P27361
YP_009725299.1,P45983
YP_009725299.1,P05412
YP_009725299.1,P04049
YP_009725299.1,P49768
YP_009725299.1,P29350
YP_009725299.1,P20339
YP_009725299.1,P63000
YP_009725299.1,P63104
YP_009725299.1,P62834
YP_009725299.1,P10276
YP_009725299.1,P20936
YP_009725299.1,Q09028
YP_009725299.1,P63208
YP_009725299.1,P14678
YP_009725299.1,P12931
YP_009725299.1,P42224
YP_009725299.1,P42229
YP_009725299.1,Q15370
YP_009725299.1,Q15369
YP_009725299.1,P01137
YP_009725299.1,P37173
YP_009725299.1,P19438
YP_009725299.1,P20333
YP_009725299.1,P09429
YP_009725299.1,O15350
YP_009725299.1,P0CG47
YP_009725299.1,P27348
YP_009725299.1,P08670
YP_009725299.1,Q13838
YP_009725299.1,Q15788
YP_009725299.1,P10275

YP_009725299.1,P63167
YP_009725299.1,O60936
YP_009725299.1,P62877
YP_009725299.1,P11142
YP_009725299.1,P07900
YP_009725299.1,Q9NPD3
YP_009725299.1,P04792
YP_009725299.1,P62979
YP_009725299.1,P0CG48
YP_009725299.1,O43561
YP_009725299.1,P19174
YP_009725299.1,Q9P2H0
YP_009725299.1,Q9Y383
YP_009725299.1,Q04917
YP_009725299.1,P60709
YP_009725299.1,P63261
YP_009725299.1,P35080
YP_009725299.1,Q01844
YP_009725299.1,P12814
YP_009725299.1,Q9Y3C7
YP_009725299.1,O15162
YP_009725299.1,Q9BQ66
YP_009725299.1,P42574
YP_009725299.1,P48023
YP_009725299.1,P68104
YP_009725299.1,P32121
YP_009725299.1,P16220
YP_009725299.1,P21246
YP_009725299.1,P46379
YP_009725299.1,P21796
YP_009725299.1,P62136
YP_009725299.1,P21333
YP_009725299.1,Q07021
YP_009725299.1,P0DP25
YP_009725299.1,Q03135
YP_009725299.1,Q04724
YP_009725299.1,O15379
YP_009725299.1,P06241
YP_009725299.1,P07948
YP_009725299.1,Q9UM11
YP_009725299.1,O95967
YP_009725299.1,P51681
YP_009725299.1,P05556
YP_009725299.1,Q13432
YP_009725299.1,P62258
YP_009725299.1,P01100
YP_009725299.1,P02462
YP_009725299.1,Q9BQY4
YP_009725299.1,Q05516
YP_009725299.1,P11831
YP_009725299.1,P04150
YP_009725299.1,Q92731
YP_009725299.1,Q7L5N1
YP_009725299.1,Q14192

YP_009725299.1,P04406
YP_009725299.1,P68133
YP_009725299.1,P19793
YP_009725299.1,P0DMV9
YP_009725299.1,O00560
YP_009725299.1,O60383
YP_009725299.1,P49023
YP_009725299.1,P15121
YP_009725299.1,P30153
YP_009725299.1,Q9NRR5
YP_009725299.1,P61981
YP_009725299.1,P27695
YP_009725299.1,P28482
YP_009725299.1,P62987
YP_009725299.1,Q9Y265
YP_009725299.1,P15514
YP_009725299.1,Q15834
YP_009725301.1,P63165
YP_009725301.1,P60953
YP_009725301.1,P04637
YP_009725301.1,P10415
YP_009725301.1,P62993
YP_009725301.1,P38936
YP_009725301.1,Q8N726
YP_009725301.1,P62826
YP_009725301.1,P63279
YP_009725301.1,P20226
YP_009725301.1,P63000
YP_009725301.1,P0CG47
YP_009725301.1,P0CG48
YP_009725301.1,Q9BQ66
YP_009725301.1,P21246
YP_009725301.1,P0DP25
YP_009725301.1,Q7L5N1
YP_009725301.1,Q9NRR5
YP_009725305.1,Q9BQ66
YP_009725305.1,P21246
YP_009725307.1,P27986
YP_009725307.1,P46108
YP_009725307.1,P06400
YP_009725307.1,P49841
YP_009725307.1,P55211
YP_009725307.1,Q12933
YP_009725307.1,P25445
YP_009725307.1,P63165
YP_009725307.1,Q13158
YP_009725307.1,Q14790
YP_009725307.1,P61586
YP_009725307.1,P60953
YP_009725307.1,P04637
YP_009725307.1,Q07817
YP_009725307.1,P10415
YP_009725307.1,P55957
YP_009725307.1,P24385

YP_009725307.1,P31946
YP_009725307.1,P22681
YP_009725307.1,P00533
YP_009725307.1,P62993
YP_009725307.1,P24941
YP_009725307.1,P11802
YP_009725307.1,P30281
YP_009725307.1,Q13114
YP_009725307.1,Q9Y4K3
YP_009725307.1,P42768
YP_009725307.1,P38936
YP_009725307.1,Q8N726
YP_009725307.1,P17676
YP_009725307.1,Q13485
YP_009725307.1,P62826
YP_009725307.1,Q92793
YP_009725307.1,P46109
YP_009725307.1,P67870
YP_009725307.1,P35222
YP_009725307.1,P63279
YP_009725307.1,Q01094
YP_009725307.1,P08047
YP_009725307.1,P26641
YP_009725307.1,P29353
YP_009725307.1,P40763
YP_009725307.1,P04626
YP_009725307.1,Q13541
YP_009725307.1,Q09472
YP_009725307.1,Q04206
YP_009725307.1,P12956
YP_009725307.1,Q06124
YP_009725307.1,P20226
YP_009725307.1,Q13547
YP_009725307.1,Q92769
YP_009725307.1,P01112
YP_009725307.1,P03372
YP_009725307.1,P63244
YP_009725307.1,Q15628
YP_009725307.1,P06239
YP_009725307.1,Q15796
YP_009725307.1,P84022
YP_009725307.1,P01106
YP_009725307.1,P16333
YP_009725307.1,P12004
YP_009725307.1,P27361
YP_009725307.1,P45983
YP_009725307.1,P05412
YP_009725307.1,P04049
YP_009725307.1,P49768
YP_009725307.1,P20339
YP_009725307.1,P63000
YP_009725307.1,P63104
YP_009725307.1,P62834
YP_009725307.1,P10276

YP_009725307.1,P20936
YP_009725307.1,P63208
YP_009725307.1,P14678
YP_009725307.1,P12931
YP_009725307.1,Q15370
YP_009725307.1,Q15369
YP_009725307.1,P19438
YP_009725307.1,P09429
YP_009725307.1,O15350
YP_009725307.1,P0CG47
YP_009725307.1,P27348
YP_009725307.1,P08670
YP_009725307.1,P10275
YP_009725307.1,P63167
YP_009725307.1,O60936
YP_009725307.1,P62877
YP_009725307.1,Q9NPD3
YP_009725307.1,P04792
YP_009725307.1,P62979
YP_009725307.1,P0CG48
YP_009725307.1,O43561
YP_009725307.1,Q9P2H0
YP_009725307.1,Q04917
YP_009725307.1,P60709
YP_009725307.1,P63261
YP_009725307.1,P35080
YP_009725307.1,Q01844
YP_009725307.1,Q9Y3C7
YP_009725307.1,O15162
YP_009725307.1,Q9BQ66
YP_009725307.1,P42574
YP_009725307.1,O75340
YP_009725307.1,P68104
YP_009725307.1,P16220
YP_009725307.1,P21246
YP_009725307.1,P46379
YP_009725307.1,P21333
YP_009725307.1,P0DP25
YP_009725307.1,Q03135
YP_009725307.1,Q04724
YP_009725307.1,P06241
YP_009725307.1,P07948
YP_009725307.1,O95967
YP_009725307.1,P05556
YP_009725307.1,Q13432
YP_009725307.1,P62258
YP_009725307.1,Q9BQY4
YP_009725307.1,Q05516
YP_009725307.1,P04150
YP_009725307.1,Q92731
YP_009725307.1,Q7L5N1
YP_009725307.1,Q14192
YP_009725307.1,P04406
YP_009725307.1,P19793

YP_009725307.1,O60383
YP_009725307.1,P49023
YP_009725307.1,P30153
YP_009725307.1,Q9NRR5
YP_009725307.1,P61981
YP_009725307.1,P28482
YP_009725307.1,P62987
YP_009725307.1,P15514
YP_009725307.1,Q15834
YP_009725300.1,P63165
YP_009725300.1,P60953
YP_009725300.1,P10415
YP_009725300.1,P62993
YP_009725300.1,P38936
YP_009725300.1,Q8N726
YP_009725300.1,P46109
YP_009725300.1,P63279
YP_009725300.1,P20226
YP_009725300.1,P63000
YP_009725300.1,P0CG47
YP_009725300.1,P0CG48
YP_009725300.1,Q9BQ66
YP_009725300.1,P21246
YP_009725300.1,P0DP25
YP_009725300.1,Q9NRR5
YP_009725298.1,P63165
YP_009725298.1,P60953
YP_009725298.1,P04637
YP_009725298.1,P10415
YP_009725298.1,P62993
YP_009725298.1,P38936
YP_009725298.1,Q8N726
YP_009725298.1,P62826
YP_009725298.1,P46109
YP_009725298.1,P63279
YP_009725298.1,Q04206
YP_009725298.1,P20226
YP_009725298.1,P63244
YP_009725298.1,P63000
YP_009725298.1,P14678
YP_009725298.1,P0CG47
YP_009725298.1,P63167
YP_009725298.1,P0CG48
YP_009725298.1,Q9BQ66
YP_009725298.1,P21246
YP_009725298.1,P0DP25
YP_009725298.1,Q03135
YP_009725298.1,Q14192
YP_009725302.1,P10415
YP_009725302.1,Q9BQ66
YP_009725302.1,P21246
YP_009725306.1,Q9BQ66
YP_009725306.1,P21246
YP_009725304.1,P10415

YP_009725304.1,P38936
YP_009725304.1,Q8N726
YP_009725304.1,P63279
YP_009725304.1,P20226
YP_009725304.1,Q9BQ66
YP_009725304.1,P21246
YP_009725304.1,Q9NRR5
YP_009724394.1,Q9BQ66
YP_009725311.1,P10415
YP_009725311.1,P62993
YP_009725311.1,P63279
YP_009725311.1,P20226
YP_009725311.1,P63167
YP_009725311.1,Q9BQ66
YP_009725311.1,P21246
YP_009725311.1,Q9NRR5
YP_009725308.1,P46108
YP_009725308.1,Q12933
YP_009725308.1,P63165
YP_009725308.1,P60953
YP_009725308.1,P04637
YP_009725308.1,P10415
YP_009725308.1,P62993
YP_009725308.1,P24941
YP_009725308.1,P42768
YP_009725308.1,P38936
YP_009725308.1,Q8N726
YP_009725308.1,P62826
YP_009725308.1,P46109
YP_009725308.1,P67870
YP_009725308.1,P35222
YP_009725308.1,P63279
YP_009725308.1,P08047
YP_009725308.1,P29353
YP_009725308.1,Q09472
YP_009725308.1,Q04206
YP_009725308.1,P20226
YP_009725308.1,P03372
YP_009725308.1,P63244
YP_009725308.1,P84022
YP_009725308.1,P49768
YP_009725308.1,P63000
YP_009725308.1,P10276
YP_009725308.1,P14678
YP_009725308.1,P0CG47
YP_009725308.1,P63167
YP_009725308.1,P62979
YP_009725308.1,P0CG48
YP_009725308.1,Q9Y3C7
YP_009725308.1,Q9BQ66
YP_009725308.1,P21246
YP_009725308.1,P0DP25
YP_009725308.1,Q03135
YP_009725308.1,Q13432

YP_009725308.1,Q7L5N1
YP_009725308.1,Q14192
YP_009725308.1,Q9NRR5
YP_009724396.1,Q9BQ66
YP_009724396.1,P21246
YP_009724392.1,Q9BQ66
YP_009724392.1,P21246
YP_009724389.1,P27986
YP_009724389.1,P46108
YP_009724389.1,P35869
YP_009724389.1,P06400
YP_009724389.1,P31749
YP_009724389.1,P49841
YP_009724389.1,P55211
YP_009724389.1,Q12933
YP_009724389.1,P51693
YP_009724389.1,P05067
YP_009724389.1,P25445
YP_009724389.1,P63165
YP_009724389.1,Q13158
YP_009724389.1,Q14790
YP_009724389.1,P61586
YP_009724389.1,P60953
YP_009724389.1,P04637
YP_009724389.1,Q12959
YP_009724389.1,Q07817
YP_009724389.1,P10415
YP_009724389.1,P55957
YP_009724389.1,P24385
YP_009724389.1,P31946
YP_009724389.1,P22681
YP_009724389.1,P00533
YP_009724389.1,P62993
YP_009724389.1,P20248
YP_009724389.1,P24941
YP_009724389.1,P11802
YP_009724389.1,P30281
YP_009724389.1,Q13114
YP_009724389.1,Q9Y4K3
YP_009724389.1,P06493
YP_009724389.1,P42768
YP_009724389.1,P38936
YP_009724389.1,Q8N726
YP_009724389.1,P17676
YP_009724389.1,Q13485
YP_009724389.1,P62826
YP_009724389.1,O15111
YP_009724389.1,O14920
YP_009724389.1,P25963
YP_009724389.1,Q92793
YP_009724389.1,Q9Y6Q9
YP_009724389.1,P46109
YP_009724389.1,P67870
YP_009724389.1,P35222

YP_009724389.1,P12830
YP_009724389.1,P63279
YP_009724389.1,Q01094
YP_009724389.1,P08047
YP_009724389.1,P26641
YP_009724389.1,P29353
YP_009724389.1,P40763
YP_009724389.1,P04626
YP_009724389.1,P06730
YP_009724389.1,Q13541
YP_009724389.1,Q09472
YP_009724389.1,Q04206
YP_009724389.1,P78352
YP_009724389.1,P00734
YP_009724389.1,P13726
YP_009724389.1,P62942
YP_009724389.1,P12956
YP_009724389.1,Q06124
YP_009724389.1,P20226
YP_009724389.1,Q13547
YP_009724389.1,Q92769
YP_009724389.1,P01112
YP_009724389.1,P03372
YP_009724389.1,P63244
YP_009724389.1,Q99836
YP_009724389.1,P05106
YP_009724389.1,Q14974
YP_009724389.1,Q15628
YP_009724389.1,P06239
YP_009724389.1,Q15796
YP_009724389.1,P84022
YP_009724389.1,P49736
YP_009724389.1,P33993
YP_009724389.1,Q00987
YP_009724389.1,P01106
YP_009724389.1,P16333
YP_009724389.1,P19838
YP_009724389.1,P12004
YP_009724389.1,P28340
YP_009724389.1,P27361
YP_009724389.1,P45983
YP_009724389.1,P05412
YP_009724389.1,Q02750
YP_009724389.1,P04049
YP_009724389.1,P49768
YP_009724389.1,Q05397
YP_009724389.1,P29350
YP_009724389.1,P20339
YP_009724389.1,P63000
YP_009724389.1,P63104
YP_009724389.1,P62834
YP_009724389.1,P10276
YP_009724389.1,P20936
YP_009724389.1,Q14686

YP_009724389.1,Q09028
YP_009724389.1,P62913
YP_009724389.1,P23246
YP_009724389.1,P63208
YP_009724389.1,Q16637
YP_009724389.1,P14678
YP_009724389.1,P12931
YP_009724389.1,P42224
YP_009724389.1,P42229
YP_009724389.1,P51692
YP_009724389.1,Q00403
YP_009724389.1,Q15370
YP_009724389.1,Q15369
YP_009724389.1,P01137
YP_009724389.1,P37173
YP_009724389.1,P19438
YP_009724389.1,P20333
YP_009724389.1,P09429
YP_009724389.1,O15350
YP_009724389.1,P0CG47
YP_009724389.1,P27348
YP_009724389.1,Q9Y6K9
YP_009724389.1,O75928
YP_009724389.1,P08670
YP_009724389.1,Q13838
YP_009724389.1,Q15788
YP_009724389.1,P10275
YP_009724389.1,P63167
YP_009724389.1,O60936
YP_009724389.1,P62877
YP_009724389.1,P11142
YP_009724389.1,Q15596
YP_009724389.1,P07900
YP_009724389.1,Q9NPD3
YP_009724389.1,P04792
YP_009724389.1,P62979
YP_009724389.1,P0CG48
YP_009724389.1,O43561
YP_009724389.1,P19174
YP_009724389.1,Q9P2H0
YP_009724389.1,Q9Y383
YP_009724389.1,Q04917
YP_009724389.1,P60709
YP_009724389.1,P63261
YP_009724389.1,P35080
YP_009724389.1,Q01844
YP_009724389.1,P12814
YP_009724389.1,O14908
YP_009724389.1,Q9Y3C7
YP_009724389.1,Q15078
YP_009724389.1,P35609
YP_009724389.1,O15162
YP_009724389.1,Q9BQ66
YP_009724389.1,P42574

YP_009724389.1,P48023
YP_009724389.1,O75340
YP_009724389.1,P68104
YP_009724389.1,P32121
YP_009724389.1,Q15047
YP_009724389.1,P18846
YP_009724389.1,P16220
YP_009724389.1,P21246
YP_009724389.1,P46379
YP_009724389.1,Q99689
YP_009724389.1,P21796
YP_009724389.1,P62136
YP_009724389.1,P21333
YP_009724389.1,Q07021
YP_009724389.1,P0DP25
YP_009724389.1,Q03135
YP_009724389.1,Q01196
YP_009724389.1,Q04724
YP_009724389.1,O15379
YP_009724389.1,P68036
YP_009724389.1,P06241
YP_009724389.1,P07948
YP_009724389.1,Q9UM11
YP_009724389.1,O95967
YP_009724389.1,P51681
YP_009724389.1,P05556
YP_009724389.1,Q13432
YP_009724389.1,P62258
YP_009724389.1,P49715
YP_009724389.1,P01100
YP_009724389.1,P36897
YP_009724389.1,P02462
YP_009724389.1,Q9BQY4
YP_009724389.1,P41240
YP_009724389.1,Q13363
YP_009724389.1,O60674
YP_009724389.1,Q05516
YP_009724389.1,Q9NP97
YP_009724389.1,P11831
YP_009724389.1,P24666
YP_009724389.1,P04150
YP_009724389.1,Q92731
YP_009724389.1,Q7L5N1
YP_009724389.1,Q14192
YP_009724389.1,P04406
YP_009724389.1,P68133
YP_009724389.1,Q99750
YP_009724389.1,P41235
YP_009724389.1,Q13526
YP_009724389.1,P19793
YP_009724389.1,P0DMV9
YP_009724389.1,P11021
YP_009724389.1,P01344
YP_009724389.1,O00560

YP_009724389.1,O60383
YP_009724389.1,P49023
YP_009724389.1,P15498
YP_009724389.1,P25791
YP_009724389.1,Q15797
YP_009724389.1,P15121
YP_009724389.1,P30153
YP_009724389.1,Q9UMX0
YP_009724389.1,Q9NRR5
YP_009724389.1,P61981
YP_009724389.1,P14174
YP_009724389.1,P27695
YP_009724389.1,P17252
YP_009724389.1,P28482
YP_009724389.1,Q15398
YP_009724389.1,P62987
YP_009724389.1,Q9Y265
YP_009724389.1,Q9Y230
YP_009724389.1,P54253
YP_009724389.1,P10114
YP_009724389.1,P15514
YP_009724389.1,Q15834
YP_009724390.1,P46108
YP_009724390.1,Q12933
YP_009724390.1,P63165
YP_009724390.1,P60953
YP_009724390.1,P04637
YP_009724390.1,P10415
YP_009724390.1,P31946
YP_009724390.1,P62993
YP_009724390.1,P38936
YP_009724390.1,Q8N726
YP_009724390.1,P62826
YP_009724390.1,P46109
YP_009724390.1,P63279
YP_009724390.1,P29353
YP_009724390.1,Q09472
YP_009724390.1,Q04206
YP_009724390.1,P20226
YP_009724390.1,P01112
YP_009724390.1,P63244
YP_009724390.1,P84022
YP_009724390.1,P16333
YP_009724390.1,P63000
YP_009724390.1,P14678
YP_009724390.1,P19438
YP_009724390.1,P0CG47
YP_009724390.1,P27348
YP_009724390.1,P63167
YP_009724390.1,P62979
YP_009724390.1,P0CG48
YP_009724390.1,Q9Y3C7
YP_009724390.1,Q9BQ66
YP_009724390.1,P42574

YP_009724390.1,P21246
YP_009724390.1,P0DP25
YP_009724390.1,Q03135
YP_009724390.1,O95967
YP_009724390.1,Q7L5N1
YP_009724390.1,Q14192
YP_009724390.1,Q9NRR5
YP_009724390.1,P62987
YP_009725310.1,P63165
YP_009725310.1,P60953
YP_009725310.1,P04637
YP_009725310.1,P10415
YP_009725310.1,P62993
YP_009725310.1,P38936
YP_009725310.1,Q8N726
YP_009725310.1,P62826
YP_009725310.1,P46109
YP_009725310.1,P63279
YP_009725310.1,Q04206
YP_009725310.1,P20226
YP_009725310.1,P63000
YP_009725310.1,P0CG47
YP_009725310.1,P63167
YP_009725310.1,P0CG48
YP_009725310.1,Q9Y3C7
YP_009725310.1,Q9BQ66
YP_009725310.1,P21246
YP_009725310.1,P0DP25
YP_009725310.1,Q03135
YP_009725310.1,Q7L5N1
YP_009725310.1,Q9NRR5
YP_009724395.1,Q9BQ66
YP_009724395.1,P21246
YP_009725309.1,P63165
YP_009725309.1,P60953
YP_009725309.1,P10415
YP_009725309.1,P62993
YP_009725309.1,P38936
YP_009725309.1,Q8N726
YP_009725309.1,P63279
YP_009725309.1,P20226
YP_009725309.1,P0CG47
YP_009725309.1,P0CG48
YP_009725309.1,Q9BQ66
YP_009725309.1,P21246
YP_009725309.1,P0DP25
YP_009724397.2,P46108
YP_009724397.2,P63165
YP_009724397.2,P60953
YP_009724397.2,P04637
YP_009724397.2,P10415
YP_009724397.2,P62993
YP_009724397.2,P42768
YP_009724397.2,P38936

YP_009724397.2,Q8N726
YP_009724397.2,P62826
YP_009724397.2,P46109
YP_009724397.2,P63279
YP_009724397.2,P20226
YP_009724397.2,P84022
YP_009724397.2,P63000
YP_009724397.2,P14678
YP_009724397.2,P0CG47
YP_009724397.2,P63167
YP_009724397.2,P0CG48
YP_009724397.2,Q9Y3C7
YP_009724397.2,Q9BQ66
YP_009724397.2,P21246
YP_009724397.2,P0DP25
YP_009724397.2,Q9UKR5
YP_009724397.2,Q9NRR5
YP_009725255.1,Q9BQ66
YP_009725255.1,P21246
YP_009725295.1,P27986
YP_009725295.1,P46108
YP_009725295.1,P35869
YP_009725295.1,P06400
YP_009725295.1,P31749
YP_009725295.1,P49841
YP_009725295.1,P55211
YP_009725295.1,Q12933
YP_009725295.1,P51693
YP_009725295.1,P05067
YP_009725295.1,P25445
YP_009725295.1,P63165
YP_009725295.1,Q13158
YP_009725295.1,Q14790
YP_009725295.1,P61586
YP_009725295.1,P60953
YP_009725295.1,P04637
YP_009725295.1,Q12959
YP_009725295.1,Q07817
YP_009725295.1,P10415
YP_009725295.1,P55957
YP_009725295.1,P24385
YP_009725295.1,P31946
YP_009725295.1,P22681
YP_009725295.1,P00533
YP_009725295.1,P62993
YP_009725295.1,P20248
YP_009725295.1,P24941
YP_009725295.1,P11802
YP_009725295.1,P30281
YP_009725295.1,Q13114
YP_009725295.1,Q9Y4K3
YP_009725295.1,P06493
YP_009725295.1,P42768
YP_009725295.1,P38936

YP_009725295.1,Q8N726
YP_009725295.1,P17676
YP_009725295.1,Q13485
YP_009725295.1,P62826
YP_009725295.1,O14920
YP_009725295.1,P25963
YP_009725295.1,Q92793
YP_009725295.1,Q9Y6Q9
YP_009725295.1,P46109
YP_009725295.1,P67870
YP_009725295.1,P35222
YP_009725295.1,P63279
YP_009725295.1,Q01094
YP_009725295.1,P08047
YP_009725295.1,P26641
YP_009725295.1,P29353
YP_009725295.1,P40763
YP_009725295.1,P04626
YP_009725295.1,P06730
YP_009725295.1,Q13541
YP_009725295.1,Q09472
YP_009725295.1,Q04206
YP_009725295.1,P78352
YP_009725295.1,P00734
YP_009725295.1,P13726
YP_009725295.1,P05230
YP_009725295.1,P62942
YP_009725295.1,P12956
YP_009725295.1,Q06124
YP_009725295.1,P20226
YP_009725295.1,Q13547
YP_009725295.1,Q92769
YP_009725295.1,P01112
YP_009725295.1,P03372
YP_009725295.1,P63244
YP_009725295.1,Q99836
YP_009725295.1,P05106
YP_009725295.1,Q14974
YP_009725295.1,Q15628
YP_009725295.1,P06239
YP_009725295.1,Q15796
YP_009725295.1,P84022
YP_009725295.1,P33993
YP_009725295.1,Q00987
YP_009725295.1,P01106
YP_009725295.1,P16333
YP_009725295.1,P19838
YP_009725295.1,P12004
YP_009725295.1,P28340
YP_009725295.1,P27361
YP_009725295.1,P45983
YP_009725295.1,P05412
YP_009725295.1,Q02750
YP_009725295.1,P04049

YP_009725295.1,P49768
YP_009725295.1,Q05397
YP_009725295.1,P29350
YP_009725295.1,P20339
YP_009725295.1,P63000
YP_009725295.1,P63104
YP_009725295.1,P62834
YP_009725295.1,P10276
YP_009725295.1,P20936
YP_009725295.1,Q09028
YP_009725295.1,P62913
YP_009725295.1,P63208
YP_009725295.1,P14678
YP_009725295.1,P12931
YP_009725295.1,P42224
YP_009725295.1,P42229
YP_009725295.1,Q15370
YP_009725295.1,Q15369
YP_009725295.1,P01137
YP_009725295.1,P37173
YP_009725295.1,P19438
YP_009725295.1,P20333
YP_009725295.1,P09429
YP_009725295.1,O15350
YP_009725295.1,P0CG47
YP_009725295.1,P27348
YP_009725295.1,P08670
YP_009725295.1,Q13838
YP_009725295.1,Q15788
YP_009725295.1,P10275
YP_009725295.1,P63167
YP_009725295.1,O60936
YP_009725295.1,P62877
YP_009725295.1,P11142
YP_009725295.1,Q15596
YP_009725295.1,P07900
YP_009725295.1,Q9NPD3
YP_009725295.1,P04792
YP_009725295.1,P62979
YP_009725295.1,P0CG48
YP_009725295.1,O43561
YP_009725295.1,P19174
YP_009725295.1,Q9P2H0
YP_009725295.1,Q9Y383
YP_009725295.1,Q04917
YP_009725295.1,P60709
YP_009725295.1,P63261
YP_009725295.1,P35080
YP_009725295.1,Q01844
YP_009725295.1,P12814
YP_009725295.1,O14908
YP_009725295.1,Q9Y3C7
YP_009725295.1,O15162
YP_009725295.1,Q9BQ66

YP_009725295.1,P42574
YP_009725295.1,P48023
YP_009725295.1,O75340
YP_009725295.1,P68104
YP_009725295.1,P32121
YP_009725295.1,Q15047
YP_009725295.1,P18846
YP_009725295.1,P16220
YP_009725295.1,P21246
YP_009725295.1,P46379
YP_009725295.1,Q99689
YP_009725295.1,P21796
YP_009725295.1,P62136
YP_009725295.1,P21333
YP_009725295.1,Q07021
YP_009725295.1,P0DP25
YP_009725295.1,Q03135
YP_009725295.1,Q04724
YP_009725295.1,O15379
YP_009725295.1,P68036
YP_009725295.1,P06241
YP_009725295.1,P07948
YP_009725295.1,Q9UM11
YP_009725295.1,O95967
YP_009725295.1,P51681
YP_009725295.1,P05556
YP_009725295.1,Q13432
YP_009725295.1,P62258
YP_009725295.1,P49715
YP_009725295.1,P01100
YP_009725295.1,P02462
YP_009725295.1,Q9BQY4
YP_009725295.1,P41240
YP_009725295.1,Q13363
YP_009725295.1,Q05516
YP_009725295.1,Q9NP97
YP_009725295.1,P11831
YP_009725295.1,P24666
YP_009725295.1,P04150
YP_009725295.1,Q92731
YP_009725295.1,Q7L5N1
YP_009725295.1,Q14192
YP_009725295.1,P04406
YP_009725295.1,P68133
YP_009725295.1,Q13526
YP_009725295.1,P19793
YP_009725295.1,P0DMV9
YP_009725295.1,P11021
YP_009725295.1,P01344
YP_009725295.1,O00560
YP_009725295.1,O60383
YP_009725295.1,P49023
YP_009725295.1,P15498
YP_009725295.1,P25791

YP_009725295.1,Q15797
YP_009725295.1,P15121
YP_009725295.1,P30153
YP_009725295.1,Q9UMX0
YP_009725295.1,Q9NRR5
YP_009725295.1,P61981
YP_009725295.1,P14174
YP_009725295.1,P27695
YP_009725295.1,P17252
YP_009725295.1,P28482
YP_009725295.1,Q15398
YP_009725295.1,P62987
YP_009725295.1,Q9Y265
YP_009725295.1,Q9Y230
YP_009725295.1,P10114
YP_009725295.1,P15514
YP_009725295.1,Q15834
YP_009724391.1,P10415
YP_009724391.1,P62993
YP_009724391.1,P63279
YP_009724391.1,Q9BQ66
YP_009724391.1,P21246
YP_009724393.1,Q9BQ66
YP_009724393.1,P21246
YP_009725297.1,P10415
YP_009725297.1,P62993
YP_009725297.1,P63279
YP_009725297.1,Q9BQ66
YP_009725297.1,P21246
YP_009725297.1,Q9NRR5
