# Supplementary material for: A new framework for host-pathogen interaction research
Source: Front Immunol. 2022 Dec 15;13:1066733. doi: 10.3389/fimmu.2022.1066733 (PMC9797517; doi:10.3389/fimmu.2022.1066733)
Supplement: Supplementary file 1 [file DataSheet_1.zip › New folder (2)/Supplemental File 4.PDF]

```
"ID","Label","Group","tissue_use"
"YP_009724389.1","YP_009724389.1",1,"virus"
"YP_009724390.1","YP_009724390.1",1,"virus"
"YP_009724391.1","YP_009724391.1",1,"virus"
"YP_009724392.1","YP_009724392.1",1,"virus"
"YP_009724393.1","YP_009724393.1",1,"virus"
"YP_009724394.1","YP_009724394.1",1,"virus"
"YP_009724395.1","YP_009724395.1",1,"virus"
"YP_009724396.1","YP_009724396.1",1,"virus"
"YP_009724397.2","YP_009724397.2",1,"virus"
"YP_009725255.1","YP_009725255.1",1,"virus"
"YP_009725295.1","YP_009725295.1",1,"virus"
"YP_009725297.1","YP_009725297.1",1,"virus"
"YP_009725298.1","YP_009725298.1",1,"virus"
"YP_009725299.1","YP_009725299.1",1,"virus"
"YP_009725300.1","YP_009725300.1",1,"virus"
"YP_009725301.1","YP_009725301.1",1,"virus"
"YP_009725302.1","YP_009725302.1",1,"virus"
"YP_009725303.1","YP_009725303.1",1,"virus"
"YP_009725304.1","YP_009725304.1",1,"virus"
"YP_009725305.1","YP_009725305.1",1,"virus"
"YP_009725306.1","YP_009725306.1",1,"virus"
"YP_009725307.1","YP_009725307.1",1,"virus"
"YP_009725308.1","YP_009725308.1",1,"virus"
"YP_009725309.1","YP_009725309.1",1,"virus"
"YP_009725310.1","YP_009725310.1",1,"virus"
"YP_009725311.1","YP_009725311.1",1,"virus"
"YP_009725318.1","YP_009725318.1",1,"virus"
"O00560","SDCBP",2,NA
"O14908","GIPC1",2,NA
"O14920","IKBKB",2,NA
"O15111","CHUK",2,NA
"O15162","PLSCR1",2,NA
"O15350","TP73",2,"brain"
"O15379","HDAC3",2,NA
"O43561","LAT",2,"blood"
"O60383","GDF9",2,"reproductive system"
"O60674","JAK2",2,NA
"O60936","NOL3",2,"skeletal muscle"
"O75340","PDCD6",2,NA
"O75928","PIAS2",2,"reproductive system"
"O95967","EFEMP2",2,NA
"P00533","EGFR",2,"reproductive system"
"P00734","F2",2,"liver"
"P01100","FOS",2,NA
"P01106","MYC",2,NA
"P01112","HRAS",2,NA
"P01344","IGF2",2,"reproductive system"
"P02462","COL4A1",2,"reproductive system"
"P03372","ESR1",2,"reproductive system"
"P04049","RAF1",2,NA
"P04150","NR3C1",2,NA
"P04406","GAPDH",2,"skeletal muscle"
"P04626","ERBB2",2,NA
```

```
"P04637","TP53",2,"other"
"P04792","HSPB1",2,"reproductive system"
"P05067","APP",2,NA
"P05106","ITGB3",2,"gland"
"P05230","FGF1",2,"brain"
"P05412","JUN",2,NA
"P05556","ITGB1",2,NA
"P06239","LCK",2,"other"
"P06241","FYN",2,NA
"P06400","RB1",2,NA
"P06493","CDK1",2,"other"
"P06730","EIF4E",2,NA
"P07900","HSP90AA1",2,"reproductive system"
"P07948","LYN",2,"other"
"P08047","SP1",2,NA
"P08670","VIM",2,NA
"P09429","HMGB1",2,NA
"P0CG47","UBB",2,NA
"P0CG48","UBC",2,NA
"P0DMV9","HSPA1B",2,"reproductive system"
"P0DP25","CALM3",2,"brain"
"P10114","RAP2A",2,"brain"
"P10275","AR",2,"liver"
"P10276","RARA",2,NA
"P10415","BCL2",2,NA
"P11021","HSPA5",2,NA
"P11142","HSPA8",2,NA
"P11802","CDK4",2,NA
"P11831","SRF",2,NA
"P12004","PCNA",2,"other"
"P12814","ACTN1",2,NA
"P12830","CDH1",2,"intestine"
"P12931","SRC",2,NA
"P12956","XRCC6",2,NA
"P13726","F3",2,NA
"P14174","MIF",2,NA
"P14678","SNRPB",2,NA
"P15121","AKR1B1",2,"gland"
"P15498","VAV1",2,"other"
"P15514","AREG",2,"reproductive system"
"P16220","CREB1",2,NA
"P16333","NCK1",2,NA
"P17252","PRKCA",2,NA
"P17676","CEBPB",2,"skeletal muscle"
"P18846","ATF1",2,NA
"P19174","PLCG1",2,NA
"P19438","TNFRSF1A",2,NA
"P19793","RXRA",2,NA
"P19838","NFKB1",2,NA
"P20226","TBP",2,NA
"P20248","CCNA2",2,"other"
"P20333","TNFRSF1B",2,"blood"
"P20339","RAB5A",2,NA
"P20936","RASA1",2,"reproductive system"
```

"P21246","PTN",2,"gland"
"P21333","FLNA",2,NA
"P21796","VDAC1",2,"skeletal muscle"
"P22681","CBL",2,"other"
"P23246","SFPQ",2,NA
"P24385","CCND1",2,NA
"P24666","ACP1",2,NA
"P24941","CDK2",2,NA
"P25445","FAS",2,NA
"P25791","LMO2",2,NA
"P25963","NFKBIA",2,"bone marrow"
"P26641","EEF1G",2,"blood"
"P27348","YWHAQ",2,NA
"P27361","MAPK3",2,NA
"P27695","APEX1",2,NA
"P27986","PIK3R1",2,NA
"P28340","POLD1",2,NA
"P28482","MAPK1",2,"brain"
"P29350","PTPN6",2,"other"
"P29353","SHC1",2,NA
"P30153","PPP2R1A",2,NA
"P30281","CCND3",2,"other"
"P31749","AKT1",2,NA
"P31946","YWHAB",2,NA
"P32121","ARRB2",2,"other"
"P33993","MCM7",2,NA
"P35080","PFN2",2,"brain"
"P35222","CTNNB1",2,NA
"P35609","ACTN2",2,"other"
"P35869","AHR",2,"reproductive system"
"P36897","TGFBR1",2,NA
"P37173","TGFBR2",2,NA
"P38936","CDKN1A",2,NA
"P40763","STAT3",2,NA
"P41235","HNF4A",2,"intestine"
"P41240","CSK",2,"other"
"P42224","STAT1",2,NA
"P42229","STAT5A",2,NA
"P42574","CASP3",2,"blood"
"P42768","WAS",2,"other"
"P45983","MAPK8",2,NA
"P46108","CRK",2,NA
"P46109","CRKL",2,NA
"P46379","BAG6",2,NA
"P48023","FASLG",2,"blood"
"P49023","PXN",2,NA
"P49715","CEBPA",2,"reproductive system"
"P49736","MCM2",2,"other"
"P49768","PSEN1",2,NA
"P49841","GSK3B",2,NA
"P51681","CCR5",2,"blood"
"P51692","STAT5B",2,NA
"P51693","APLP1",2,"brain"
"P54253","ATXN1",2,NA

"P55211","CASP9",2,NA
"P55957","BID",2,"blood"
"P60709","ACTB",2,NA
"P60953","CDC42",2,NA
"P61586","RHOA",2,NA
"P61981","YWHAG",2,"skeletal muscle"
"P62136","PPP1CA",2,"blood"
"P62258","YWHAE",2,NA
"P62826","RAN",2,NA
"P62834","RAP1A",2,NA
"P62877","RBX1",2,NA
"P62913","RPL11",2,NA
"P62942","FKBP1A",2,NA
"P62979","RPS27A",2,NA
"P62987","UBA52",2,NA
"P62993","GRB2",2,NA
"P63000","RAC1",2,NA
"P63104","YWHAZ",2,NA
"P63165","SUMO1",2,NA
"P63167","DYNLL1",2,NA
"P63208","SKP1",2,NA
"P63244","RACK1",2,NA
"P63261","ACTG1",2,NA
"P63279","UBE2I",2,NA
"P67870","CSNK2B",2,NA
"P68036","UBE2L3",2,NA
"P68104","EEF1A1",2,NA
"P68133","ACTA1",2,"other"
"P78352","DLG4",2,"brain"
"P84022","SMAD3",2,NA
"Q00403","GTF2B",2,NA
"Q00987","MDM2",2,NA
"Q01094","E2F1",2,"bone marrow"
"Q01196","RUNX1",2,"other"
"Q01844","EWSR1",2,NA
"Q02750","MAP2K1",2,NA
"Q03135","CAV1",2,NA
"Q04206","RELA",2,NA
"Q04724","TLE1",2,NA
"Q04917","YWHAH",2,"brain"
"Q05397","PTK2",2,NA
"Q05516","ZBTB16",2,NA
"Q06124","PTPN11",2,NA
"Q07021","C1QBP",2,NA
"Q07817","BCL2L1",2,NA
"Q09028","RBBP4",2,NA
"Q09472","EP300",2,NA
"Q12933","TRAF2",2,NA
"Q12959","DLG1",2,NA
"Q13114","TRAF3",2,NA
"Q13158","FADD",2,NA
"Q13363","CTBP1",2,NA
"Q13432","UNC119",2,"other"
"Q13485","SMAD4",2,NA

"Q13526","PIN1",2,"blood"
"Q13541","EIF4EBP1",2,"gland"
"Q13547","HDAC1",2,NA
"Q13838","DDX39B",2,NA
"Q14192","FHL2",2,"reproductive system"
"Q14686","NCOA6",2,NA
"Q14790","CASP8",2,"blood"
"Q14974","KPNB1",2,NA
"Q15047","SETDB1",2,NA
"Q15078","CDK5R1",2,"brain"
"Q15369","ELOC",2,NA
"Q15370","ELOB",2,NA
"Q15398","DLGAP5",2,"other"
"Q15596","NCOA2",2,NA
"Q15628","TRADD",2,NA
"Q15788","NCOA1",2,NA
"Q15796","SMAD2",2,NA
"Q15797","SMAD1",2,NA
"Q15834","CCDC85B",2,"skeletal muscle"
"Q16637","SMN2",2,NA
"Q16637","SMN1",2,NA
"Q7L5N1","COPS6",2,NA
"Q8N726","CDKN2A",2,NA
"Q92731","ESR2",2,"reproductive system"
"Q92769","HDAC2",2,NA
"Q92793","CREBBP",2,NA
"Q99689","FEZ1",2,"brain"
"Q99750","MDFI",2,NA
"Q99836","MYD88",2,"blood"
"Q9BQ66","KRTAP4-12",2,"skin"
"Q9BQY4","RHOXF2",2,"reproductive system"
"Q9NP97","DYNLRB1",2,NA
"Q9NPD3","EXOSC4",2,NA
"Q9NRR5","UBQLN4",2,NA
"Q9P2H0","CEP126",2,"gland"
"Q9UKR5","ERG28",2,NA
"Q9UM11","FZR1",2,NA
"Q9UMX0","UBQLN1",2,NA
"Q9Y230","RUVBL2",2,"reproductive system"
"Q9Y265","RUVBL1",2,NA
"Q9Y383","LUC7L2",2,NA
"Q9Y3C7","MED31",2,NA
"Q9Y4K3","TRAF6",2,NA
"Q9Y6K9","IKBKG",2,"blood"
"Q9Y6Q9","NCOA3",2,NA
